# Holography and Coherent Diffraction Imaging with Low-(30–250 eV) and High-(80–300 keV) Energy Electrons: History, Principles, and Recent Trends

**DOI:** 10.3390/ma13143089

**Published:** 2020-07-10

**Authors:** Tatiana Latychevskaia

**Affiliations:** 1 Physics Institute, University of Zurich, Winterthurerstrasse 190, 8057 Zurich, Switzerland; tatiana@physik.uzh.ch; 2 Paul Scherrer Institute, Forschungsstrasse 111, 5232 Villigen, Switzerland

**Keywords:** holography, electron holography, in-line holography, diffraction, coherent diffraction imaging, iterative phase retrieval, biomolecules

## Abstract

In this paper, we present the theoretical background to electron scattering in an atomic potential and the differences between low- and high-energy electrons interacting with matter. We discuss several interferometric techniques that can be realized with low- and high-energy electrons and which can be applied to the imaging of non-crystalline samples and individual macromolecules, including in-line holography, point projection microscopy, off-axis holography, and coherent diffraction imaging. The advantages of using low- and high-energy electrons for particular experiments are examined, and experimental schemes for holography and coherent diffraction imaging are compared.

## 1. Introduction

We present the theoretical background to electron scattering in an atomic potential, and highlight the differences between low- and high-energy electrons interacting with matter. This theoretical introduction provides the background and definitions necessary for the remaining sections. We then present several interferometric techniques that can be realized with electrons, following the chronological order in which they were discovered (in-line holography, point projection microscopy, off-axis holography, and coherent diffraction imaging), and provide some examples. The advantages and disadvantages of various techniques realized with low- and high-energy electrons are discussed.

## 2. Electron Waves

### 2.1. The Wavelength of an Electron

In 1924, Louis de Broglie published his PhD thesis entitled “Research on the Theory of the Quanta” [1], in which he described the hypothesis that with every particle of matter with mass m and velocity *v* a real wave must be ‘associated’ and defined the wavelength

(1) $$\lambda =\frac{h}{p},$$

where h is the Planck’s constant and p is the momentum. Although de Broglie formulated his hypothesis for any type of particle, he won the Nobel Prize for Physics in 1929 for his discovery of the wave nature of electrons, after their wave-like behavior was first experimentally demonstrated in 1927 by Clinton Davisson and Lester Germer [2].

The wavelength of an accelerated electron in an electron microscope can be derived from the energy-momentum relation

(2) $$E_{\mathrm{total}}^{2}={\left(pc\right)}^{2}+{\left(m_{0}c^{2}\right)}^{2}$$

and the total energy of the electron

(3) $$E_{\mathrm{total}}=m_{0}c^{2}+eU.$$

Here, $m_{0}$ is the electron’s rest mass and eU is the kinetic energy of the electron, which arises from the accelerating voltage U. By combining Equations (2) and (3), we obtain an expression for the momentum, p

(4) $$p=\frac{1}{c}\sqrt{eU\left(2m_{0}c^{2}+eU\right)}.$$

By substituting p from Equation (4) into Equation (1) we obtain the wavelength of the electron

(5) $$\lambda =\frac{hc}{\sqrt{eU\left(2m_{0}c^{2}+eU\right)}}.$$

The values of this wavelength can range from 8.59 pm for a 20 keV electron to 1.97 pm for a 300 keV electron, where 20–300 keV is the range of energies for medium energy transmission electron microscope (TEM), which are the most widespread equipment nowadays for the study of organic and inorganic matter. 

For low-energy electrons with kinetic energies of up to 300 eV, the relativistic effects can be neglected, and the wavelength of the electron can be calculated as

(6) $$\lambda =\frac{h}{\sqrt{2m_{0}eU}}.$$

Here, the values of the wavelength range from 1.73 Å for a 50 eV electron to 0.78 Å for 250 eV. 

### 2.2. Electron Scattering in the First Born Approximation

#### 2.2.1. The Schrödinger Equation

Electrons are scattered by atomic potentials, and the wavefunction of a particle moving in a potential $V\left(\vec{r}\right)$ is described by the Schrödinger equation

(7) $$\left[-\frac{\hbar^{2}}{2m}\nabla^{2}+V\left(\vec{r}\right)\right]\psi \left(\vec{r}\right)=E\psi \left(\vec{r}\right),$$

where $\psi \left(\vec{r}\right)$ is the wavefunction of the electron, E is its energy, and ℏ is the reduced Planck’s constant $\hbar =h/\left(2\pi \right)$. The Schrödinger equation can be re-arranged as follows:

(8) $$\left(\nabla^{2}+k^{2}\right)\psi \left(\vec{r}\right)=\frac{2m}{\hbar^{2}}V\left(\vec{r}\right)\psi \left(\vec{r}\right),$$

where we have introduced $k^{2}=\frac{2m}{\hbar^{2}}E$. The solution to the Schrödinger equation (Equation (8)) can be written in the form:

(9) $$\psi \left(\vec{r}\right)=\psi_{0}\left(\vec{r}\right)+\frac{2m}{\hbar^{2}}∭G\left({\vec{r}}_{0}-\vec{r}\right)V\left({\vec{r}}_{0}\right)\psi \left({\vec{r}}_{0}\right)d{\vec{r}}_{0},$$

where $\psi_{0}\left(\vec{r}\right)$ is the solution to the homogeneous equation $\left(\nabla^{2}+k^{2}\right)\psi_{0}\left(\vec{r}\right)=0$ and $G\left(\vec{r}\right)$ is the solution to $\left(\nabla^{2}+k^{2}\right)G\left(\vec{r}\right)=\delta \left(\vec{r}\right)$. $G\left(\vec{r}\right)$ is the so-called Green function: $G^{\pm }\left(\vec{r}\right)=-\frac{1}{4\pi }\frac{e^{\pm ikr}}{r}$, which describes convergent (−) or divergent (+) spherical waves. For a stationary scattering wave, we can choose $G\left(\vec{r}\right)=G^{+}\left(\vec{r}\right)$, and can rewrite the solution to the Schrödinger equation Equation (9) as follows:

(10) $$\psi \left(\vec{r}\right)=\psi_{0}\left(\vec{r}\right)-\frac{m}{2\pi \hbar^{2}}∭\frac{e^{ik\left|\vec{r}-{\vec{r}}_{0}\right|}}{\left|\vec{r}-{\vec{r}}_{0}\right|}V\left({\vec{r}}_{0}\right)\psi \left({\vec{r}}_{0}\right)d{\vec{r}}_{0}.$$

Next, we will make use of the fact that the scattering potential $V\left(\vec{r}\right)$ is localized within a small region, meaning that $r_{0}\approx 0$. Since we are interested in the electron wavefunction far from the scattering center, we can use the approximation $r\gg r_{0}$ and can then expand

(11) $$\left|\vec{r}-{\vec{r}}_{0}\right|=\sqrt{r^{2}-2\vec{r}{\vec{r}}_{0}+r_{0}^{2}}\approx r\sqrt{1-\frac{2\vec{r}{\vec{r}}_{0}}{r^{2}}}\approx r-\frac{\vec{r}{\vec{r}}_{0}}{r}.$$

In Equation (11) and everywhere else in the text, scalar product of vectors is used, unless otherwise specified. Using the approximation in Equation (11), we rewrite Equation (10) as

(12) $$\psi \left(\vec{r}\right)=\psi_{0}\left(\vec{r}\right)-\frac{m}{2\pi \hbar^{2}}\frac{e^{ikr}}{r}∭e^{-i\vec{k}{\vec{r}}_{0}}V\left({\vec{r}}_{0}\right)\psi \left({\vec{r}}_{0}\right)d{\vec{r}}_{0}$$

where we have introduced $\vec{k}=k\frac{\vec{r}}{r}$ as the wave vector of the scattered wave. 

#### 2.2.2. The Born Approximation

Note that in Equation (12), the solution for $\psi \left(\vec{r}\right)$ contains the same function $\psi \left({\vec{r}}_{0}\right)$ in the integral. We must therefore search for a solution by applying a series of approximations. We apply the Born approximation, which originates from perturbation theory and considers only the first term of the series expansion. In the zero-th order approximation, we keep only the first term: $\psi \left(\vec{r}\right)\approx \psi_{0}\left(\vec{r}\right).$ In the first-order approximation, we use the $\psi \left(\vec{r}\right)$ found in the zero-th order approximation and substitute it into the integral given in Equation (12):

(13) $$\psi_{1}\left(\vec{r}\right)\approx \psi_{0}\left(\vec{r}\right)-\frac{m}{2\pi \hbar^{2}}\frac{e^{ikr}}{r}∭e^{-i\vec{k}{\vec{r}}_{0}}V\left({\vec{r}}_{0}\right)\psi_{0}\left({\vec{r}}_{0}\right)d{\vec{r}}_{0}.$$

In the second-order approximation, we use the $\psi_{1}\left(\vec{r}\right)$ found in the first order approximation (Equation (13)) and substitute it into the integral given by Equation (12):

(14) $$\psi_{2}\left(\vec{r}\right)\approx \psi_{0}\left(\vec{r}\right)-\frac{m}{2\pi \hbar^{2}}\frac{e^{ikr}}{r}∭e^{-i\vec{k}{\vec{r}}_{0}}V\left({\vec{r}}_{0}\right)\psi_{1}\left({\vec{r}}_{0}\right)d{\vec{r}}_{0},$$

and so forth. Typically, the first-order approximation is sufficient to describe the scattered wavefront (Equation (13)).

#### 2.2.3. Scattering Amplitude

For a plane incident wave $\psi_{0}\left(\vec{r}\right)=Ae^{ikz},$, the scattered wave in the first-order Born approximation is given by:

(15) $$\psi \left(\vec{r}\right)=Ae^{ikz}-\frac{m}{2\pi \hbar^{2}}\frac{e^{ikr}}{r}∭e^{-i\vec{k}{\vec{r}}_{0}}V\left({\vec{r}}_{0}\right)\psi_{0}\left({\vec{r}}_{0}\right)d{\vec{r}}_{0},$$

which can be re-written as

(16) $$\psi \left(\vec{r}\right)=Ae^{ikz}+f\left(\vartheta ,\varphi \right)A\frac{e^{ikr}}{r}.$$

Here, $Ae^{ikz}$ is the incident plane wave, $A\frac{e^{ikr}}{r}$ is the outgoing spherical wave (as also described by the Huygens-Fresnel principle), and $f\left(\vartheta ,\varphi \right)$ is the complex-valued scattering amplitude:

(17) $$f\left(\vartheta ,\varphi \right)=-\frac{m}{2\pi \hbar^{2}A}∭e^{-i\vec{k}{\vec{r}}_{0}}V\left({\vec{r}}_{0}\right)\psi_{0}\left({\vec{r}}_{0}\right)d{\vec{r}}_{0}.$$

**Figure 1:**
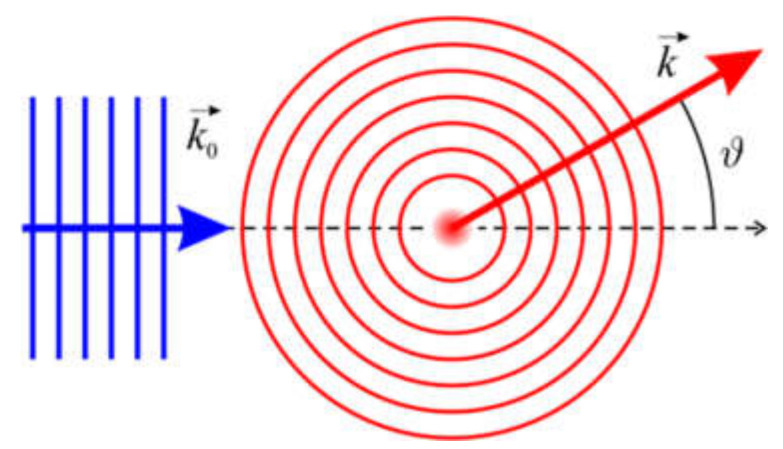
Schematic of the electron scattering event and illustration of the symbols used. ${\vec{k}}_{0}$ and $\vec{k}$ are the wave vectors of the incident plane wave and the scattered wave, respectively, and ϑ is the scattering angle.

The incident plane wave can be rewritten as $\psi_{0}\left({\vec{r}}_{0}\right)=Ae^{ikz_{0}}=Ae^{i{\vec{k}}_{0}{\vec{r}}_{0}}$, where ${\vec{k}}_{0}=k{\vec{e}}_{z}$ is the wave vector of the incident plane wave. This geometrical arrangement and the symbols used are illustrated in Figure 1. By re-writing Equation (17), we get the result that, in the first-order Born approximation, the scattering amplitude is the Fourier transform (FT) of the scattering potential:

(18) $$f\left(\vartheta ,\varphi \right)=-\frac{m}{2\pi \hbar^{2}A}∭e^{-i\left(\vec{k}-{\vec{k}}_{0}\right){\vec{r}}_{0}}V\left({\vec{r}}_{0}\right)d{\vec{r}}_{0}.$$

The differential scattering cross-section is given through the scattering amplitude as:

(19) $$\frac{d\sigma }{d\Omega }={\left|f\left(\vartheta ,\varphi \right)\right|}^{2}.$$

In Equation (19), σ is the elastic cross section, where the elastic scattering events form the signal of most of the imaging methods in TEM and also in both holographic and coherent diffraction imaging methods.

#### 2.2.4. Examples of Scattering Amplitudes

**Figure 2:**
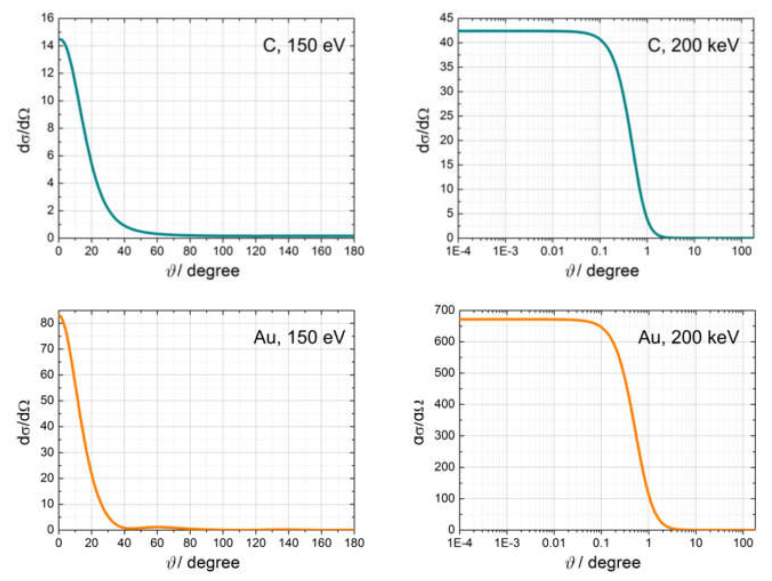
Differential scattering cross-sections dσ/dΩ of 150 eV and 200 keV electrons scattered by C and Au atoms, calculated as a function of the scattering angle ϑ. The units for the differential cross-sections are $a_{0}^{2}/\mathrm{sr}$, where $a_{0}$ is the Bohr radius.

Differential scattering cross-sections dσ/dΩ, calculated as a function of the scattering angle for carbon (C) and gold (Au) atoms, are shown in Figure 2 for electrons with energy 150 and 200 keV. The differential scattering cross-sections were calculated using the NIST Electron Elastic-Scattering Cross-Section Database (version 3.2, National Institute of Standards and Technology, Gaithersburg, MD, USA) [3]. From the plots shown in Figure 2, we can draw the following conclusions. The scattering amplitude of the electrons is maximal in the direction of the incident beam. Low-energy electrons (150 eV) scatter with a maximal amplitude within a cone of 40–60°, while high-energy electrons (200 keV) scatter with maximal amplitude within a very narrow cone of up to 1°. Elements with higher atomic numbers scatter more strongly; for example, 200 keV electrons are scattered 15 times more strongly by Au (atomic number 79) than by C (atomic number 6). 

#### 2.2.5. Inelastic Mean Free Path (IMFP) for High- and Low-Energy Electrons

**Figure 3:**
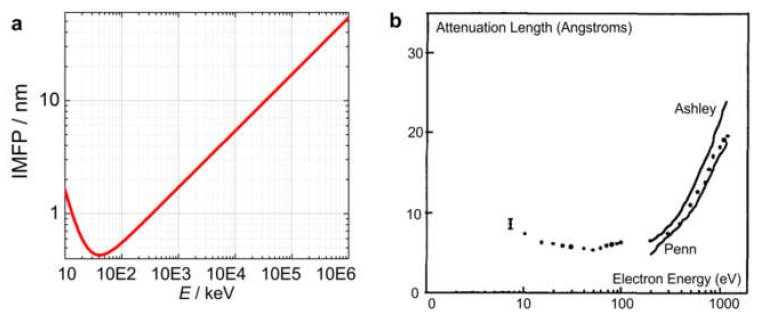
Inelastic mean free path (IMFP) as a function of electron energy. (**a**) IMFP calculated according to Equation (21). (**b**) IMFP measured based on the transmission of an electron beam through a thin amorphous carbon film as a function of the kinetic energy of the electron beam, using a transmission energy loss spectrometer [5]. Continuous lines are theoretical predictions for the IMFP by Ashley [6] and Penn [7], respectively; reprinted from [5], with permission from Elsevier.

An important difference between low- and high-energy electrons is the inelastic mean free path (IMFP), which is the average distance between inelastic scattering events. IMFP defines the maximal thickness of the samples which can be imaged with electrons. When an electron beam is propagating through a material, it loses intensity according to the expression

(20) $$I\left(z\right)=I_{0}\exp\left(-\frac{z}{\lambda_{i}}\right)$$

where $\lambda_{i}$ is the IMFP. A generalized expression for the IMFP as a function of electron energy was derived by Seah and Dench as follows [4]:

(21) $$\lambda_{i}=\frac{143}{E^{2}}+0.054\sqrt{E},$$

where E is the electron energy in eV and $\lambda_{i}$ is the IMFP in nm, as plotted in Figure 3a. The IMFP for low-energy electrons, measured experimentally using carbon films, is shown in Figure 3b. For low-energy electrons, the IMFP is on the order of a few Angstroms, which implies that only samples that are a few Angstroms thick can be measured in transmission mode. For high-energy electrons (with typical electron energies of 80–300 keV in TEMs), the IMFP ranges from tens to hundreds of nanometers, allowing us to probe thicker samples in transmission mode.

### 2.3. Transmission Function, Object Phase, Exit Wave, and Phase Problem

**Figure 4:**
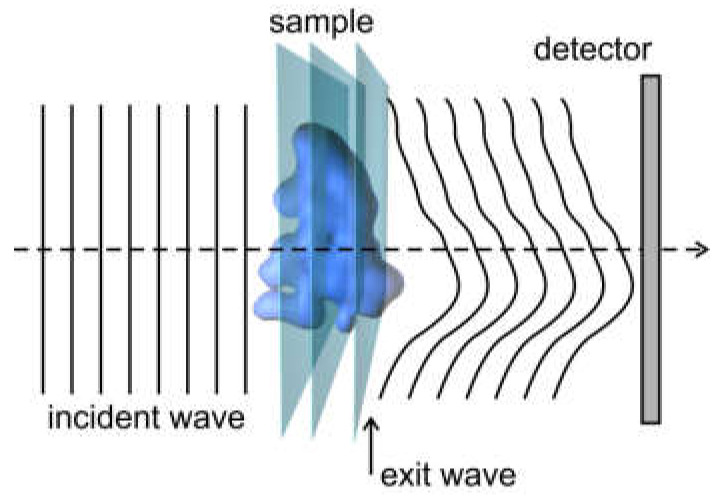
Schematic illustrating the object phase, exit wave, and phase problem.

In physics, and particularly in optics, the term “phase” is often used for a range of different phenomena. To clarify this terminology, we consider the typical arrangement of an optical experiment, as shown in Figure 4. An incident wavefront propagates through a sample and then toward a detector, where the intensity is measured. 

Each plane in the sample can be represented by a transmission function:

(22) $$t\left(x,y\right)=\exp\left[-a\left(x,y\right)\right]\exp\left[i\varphi \left(x,y\right)\right],$$

where the distribution $a\left(x,y\right)$ describes the absorbing properties of the sample and the distribution $\varphi \left(x,y\right)$ describes the phase shift introduced by the sample into the probing wave. $\varphi \left(x,y\right)$ is called the object phase. After the incident wave propagates through the entire sample, the complex-valued distribution of the wavefront immediately behind the sample is called the exit wave. The distribution of this exit wave is often reconstructed from an experimental record. Finally, the exit wave propagates toward a detector, where the distribution of the wavefront can be written as $U\left(X,Y\right)=U_{0}\left(X,Y\right)e^{i\Phi \left(X,Y\right)}$, where $U_{0}\left(X,Y\right)$ is the amplitude and $\Phi \left(X,Y\right)$ is the phase distribution. Since a detector can only record the intensity $I\left(X,Y\right)={\left|U_{0}\left(X,Y\right)\right|}^{2}$, information about the phase distribution is completely lost. However, this information is important in reconstructing the complete complex-valued wavefront in the detector plane, since the phase distribution contains information about the individual scattering events that took place inside the sample. Hence, to reconstruct the sample, we not only need to record the intensity of the diffracted wave, but must also know or determine its phase. This constitutes the phase problem.

### 2.4. Phase Shift of an Electron Wave in Electric and Magnetic Fields

When an electron is moving in an electric or magnetic potential, its wavefunction gains an additional phase shift compared to that of an electron moving through a region without a potential. In this section, we derive the phase shifts for an electron moving in both electric and magnetic potentials.

#### 2.4.1. Phase Shift of an Electron Wave in an Electric Potential

The wavefunction of an accelerated electron moving in an electric potential is described by the time-dependent Schrödinger equation:

(23) $$\left[-\frac{\hbar^{2}}{2m}\nabla^{2}+V\left(\vec{r}\right)\right]\Psi \left(\vec{r},t\right)=i\hbar \frac{\partial \Psi \left(\vec{r},t\right)}{\partial t},$$

where the time-dependent component of the eigenfunction $\Psi \left(\vec{r},t\right)$ is given by $\exp\left(-Et/\hbar \right)$. For a particle with charge q, its energy E depends on the electrostatic potential, and in a region with a constant potential V, the potential energy qV is added to E, resulting in an additional phase shift of

(24) $$\Delta \varphi =-\frac{qV}{\hbar }t,$$

where t is the time spent in the potential. For a region with potential $V\left(x,y,z\right)$, the phase shift of an electron q=−e moving along the *z*-direction is given by:

(25) $$\Delta \varphi_{E}=\frac{e}{\hbar v}\int_{\mathrm{path}}V\left(x,y,z\right)dz=\frac{e}{\hbar v}V_{z}\left(x,y\right)=\sigma V_{z}\left(x,y\right),$$

where v is the velocity of the electron, and $V_{z}\left(x,y\right)$ is the potential $V\left(\vec{r}\right)$ projected on the $\left(x,y\right)$ plane:

(26) $$V_{z}\left(x,y\right)=\int V\left(x,y,z\right)dz.$$

In Equation (25), we introduced the interaction parameter

(27) $$\sigma =\frac{e}{\hbar v}.$$

#### 2.4.2. Transmission Functions

**Figure 5:**
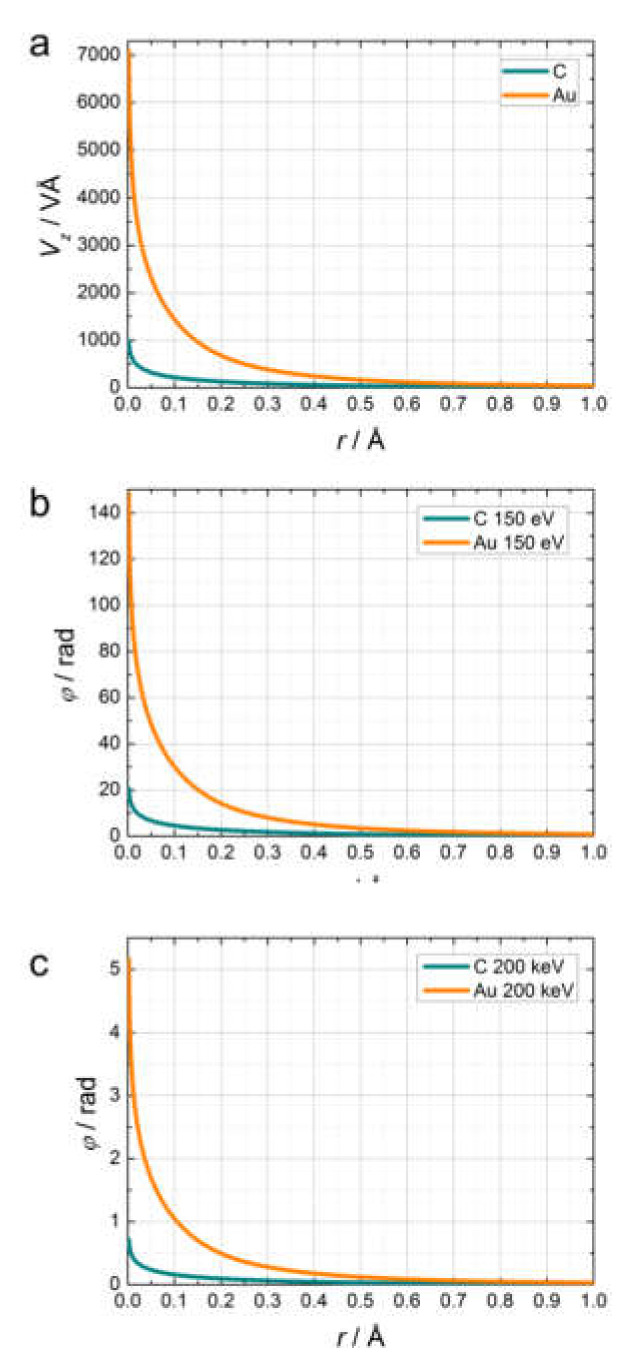
Calculated projected potentials for C and Au atoms (**a**), relative phase shifts introduced by C and Au atoms when probed with 150 eV (**b**), and 200 keV (**c**) electrons.

Figure 5a shows the projected potentials for C and Au atoms, which were calculated using the parameterized atomic potentials, as explained in reference [8]. The results in Figure 5a show that the Au atom has a much stronger projected potential than the C atom, and as a result introduces a much stronger phase shift. The phase shifts for the 150 eV and 200 keV electrons used to probe the C and Au atoms, calculated by Equation (25), are shown in Figure 5b,c, respectively. The phase shift depends on the projected potential and the interaction parameter. The interaction parameter is larger for low-energy electrons and it is relatively small for high-energy electrons [9]. For electrons of energy 200 keV, the estimated phase shift at r = 0.1 Å is 1.04 rad for Au and 0.16 rad for C. Thus, when imaged with 200 keV electrons, the C atom can be considered a weak phase object. The transmission function of a weak phase object can be approximated as

(28) $$t\left(x,y\right)=\exp\left[i\sigma V_{z}\left(x,y\right)\right]\approx 1+\sigma V_{z}\left(x,y\right).$$

#### 2.4.3. Phase Shift of an Electron Wave in a Magnetic Potential

For a charged particle moving in a magnetic potential, the total momentum is given by:

(29) $$\vec{p}=\frac{m\vec{v}}{\sqrt{1-v^{2}/c^{2}}}+q\vec{A}.$$

This momentum is preserved during the movement of the particle in a magnetic potential. The phase shift of an electron moving in a magnetic potential can be written in the form

(30) $$\varphi_{M}\left(x,y\right)=-\frac{e}{\hbar }\int_{-\infty }^{+\infty }A_{z}\left(x,y\right)dz.$$

The phase difference between two arbitrary points at coordinates $\left(x_{1},y_{1}\right)$ and $\left(x_{2},y_{2}\right)$ can be written in the form of a loop integral

(31) $$\Delta \varphi_{M}=\varphi_{M}\left(x_{1},y_{1}\right)-\varphi_{M}\left(x_{2},y_{2}\right)=-\frac{e}{\hbar }\oint \vec{A}d\vec{l}$$

for a rectangular loop formed by two parallel electron trajectories crossing the sample at coordinates $\left(x_{1},y_{1}\right)$ and $\left(x_{2},y_{2}\right)$ and joined, at infinity, by segments perpendicular to the trajectories [10]. By applying Stoke’s theorem, the phase shift can be expressed through the magnetic flux:

(32) $$\Delta \varphi_{M}=\frac{e}{\hbar }\int \vec{B}d\vec{S}=\frac{e}{\hbar }\Phi_{M},$$

where $\Phi_{M}$ is the magnetic flux through the whole region of space bounded by two electron trajectories crossing the sample at the positions of these two points.

### 2.5. Wavefront Propagation: Fresnel and Fraunhofer Diffraction

**Figure 6:**
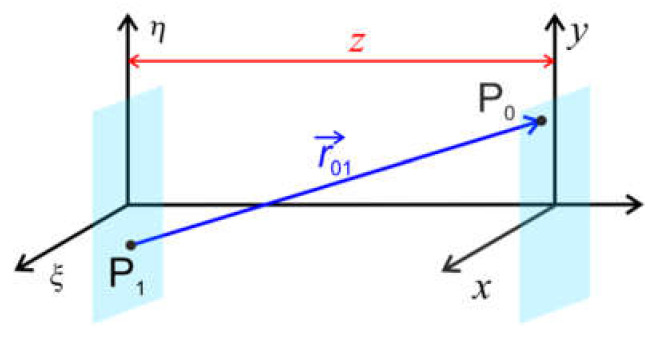
Schematic of symbols used in the Huygens-Fresnel principle, and the Fresnel and Fraunhofer diffraction integrals.

The propagation of electron waves can be described by the diffraction theory. We suppose that the complex-valued wavefront distribution is known at some plane $\left(\xi ,\eta \right)$. The propagation of a complex-valued wavefront to a point P_0_ in the plane $\left(x,y\right)$ can be calculated by employing the Huygens-Fresnel principle [11]:

(33) $$u_{0}\left(P_{0}\right)=-\frac{i}{\lambda }\iint_{S}u_{1}\left(P_{1}\right)\frac{e^{ikr_{01}}}{r_{01}}dS,$$

where P_1_ is a point in the plane $\left(\xi ,\eta \right)$, $r_{01}$ is the distance between points P_0_ and P_1_, and the integration is performed over the entire plane $\left(\xi ,\eta \right)$, as illustrated in Figure 6.

$r_{01}$ can be written using the Taylor series, as follows:

(34) $$r_{01}=\sqrt{{\left(x-\xi \right)}^{2}+{\left(y-\eta \right)}^{2}+z^{2}}\approx z+\frac{{\left(x-\xi \right)}^{2}+{\left(y-\eta \right)}^{2}}{2z},$$

provided that

(35) $$z^{3}\gg \frac{\pi }{4\lambda }{\left[{\left(x-\xi \right)}^{2}+{\left(y-\eta \right)}^{2}\right]}_{\max}^{2}.$$

At a distance z which satisfies Equation (35), the Fresnel diffraction regime is observed, and the diffracted wavefront is described by

(36) $$U_{0}\left(x,y\right)\approx -\frac{i}{\lambda z}e^{ikz}\iint_{S}U_{1}\left(\xi ,\eta \right)e^{ik\frac{{\left(x-\xi \right)}^{2}+{\left(y-\eta \right)}^{2}}{2z}}d\xi d\eta ,$$

which is obtained by substituting Equation (34) into Equation (33). From Equation (36), we see that in the Fresnel diffraction regime, the distribution of the propagated wave is simply given by a convolution of the original wave distribution with the free space propagation function $e^{ik\frac{x^{2}+y^{2}}{2z}}$.

At even larger z distances, such that

(37) $$z\gg \frac{\pi }{\lambda }{\left[\xi^{2}+\eta^{2}\right]}_{\max},$$

a Taylor series expansion gives

(38) $$r_{01}\approx z+\frac{\left(x^{2}-2x\xi \right)+(y^{2}-2y\eta )}{2z},$$

and by substituting Equation (38) into Equation (33), we obtain the wavefront distribution in the Fraunhofer diffraction regime

(39) $$U_{0}\left(x,y\right)\approx -\frac{i}{\lambda z}e^{ikz}e^{ik\frac{x^{2}+y^{2}}{2z}}\iint_{S}U_{1}\left(\xi ,\eta \right)e^{-\frac{2\pi i}{\lambda z}\left(x\xi +y\eta \right)}d\xi d\eta ,$$

which is just a two-dimensional (2D) FT of the wavefront distribution at the plane $\left(\xi ,\eta \right)$.

## 3. Holography Principle

In general, holography can be described as a measurement technique in which a known signal is superimposed with an unknown signal, and the latter can then be unambiguously reconstructed from the interference pattern that is created. For two interfering waves, this principle can be mathematically written as the holographic equation:

(40) $$H={\left|U_{R}+U_{O}\right|}^{2}={\left|U_{R}\right|}^{2}+{\left|U_{O}\right|}^{2}+U_{R}^{*}U_{O}+U_{R}U_{O}^{*},$$

where $U_{R}$ is the reference wave, $U_{O}$ is the object (unknown) wave, and H is the resulting hologram. In Equation (40), the first term ${\left|U_{R}\right|}^{2}$ is a constant distribution associated with the background, which is obtained without the presence of the object. The second term ${\left|U_{O}\right|}^{2}$ is assumed to be small, and can be neglected. The third and fourth terms are the object and twin image terms, $U_{R}^{*}U_{O}$ and $U_{R}U_{O}^{*}$, respectively, which create the interference pattern. From the holographic equation, it follows that provided H and $U_{R}$ are known, $U_{O}$ can be reconstructed as $U_{R}H\propto U_{O}+U_{R}^{2}U_{O}^{*}$. However, there will be always a remaining signal from the conjugated twin image term, $U_{R}^{2}U_{O}^{*}$.

## 4. Coherence

All imaging techniques that employ an interference pattern (for example holography or coherent diffractive imaging (CDI)) require coherent waves. Coherence characterizes the stability of the phase difference between two interfering waves. The contrast of an interference pattern is given by the coherence of the interfering waves. The visibility (contrast) of the interference pattern gives the degree of coherence [12,13]. Probing radiation is characterized by temporal (longitudinal) and spatial (transverse) coherence. 

Temporal (longitudinal) coherence is a measure of how monochromatic a source is. The temporal coherence length $l_{c}^{\mathrm{Temporal}}$ of a source with wavelength spread λ±Δλ is given by $l_{c}^{\mathrm{Temporal}}\approx \lambda^{2}/\Delta \lambda$. For example, $l_{c}^{\mathrm{Temporal}}\approx$ 390 nm for low-energy electrons with energy 250 ± 0.1 eV, and $l_{c}^{\mathrm{Temporal}}\approx$ 1 μm for high-energy electrons with energy 200,000 ± 1 eV. In both cases, the temporal coherence length exceeds the sizes of the objects typically studied in electron microscopy.

Spatial (transverse) coherence is defined by the size of the virtual source. According to the van Cittert-Zernike theorem [14,15], the complex coherence factor is given by the FT of the intensity distribution of the source [16]. For a source with intensity distribution described by a Gaussian function $s\left(\xi ,\eta \right)=\exp\left(-\frac{\xi^{2}+\eta^{2}}{2\sigma^{2}}\right)$, where $\left(\xi ,\eta \right)$ are the coordinates in the source plane and σ is the standard deviation, the spatial coherence length at a distance L from the source is given by $l_{c}^{\mathrm{Spatial}}=\frac{\lambda L}{2\pi \sigma }$ [17]. The spatial coherence is inversely proportional to the source size. For example, for low-energy electrons of energy 250 eV (wavelength = 0.078 nm), and a virtual source with σ=1 Å, the coherence length amounts to about 120 nm at a distance of L=1 μm from the source, which is sufficient to image a macromolecule of a size of few tens of nanometers. For high-energy electrons of energy 200 keV (wavelength = 2.51 pm), and a virtual source with σ=1 Å, the coherence length amounts to about 4 nm at a distance of L=1 μm from the source. It must be noted that in the case of high-energy electrons employed in a TEM, the spatial coherence length depends not only on the source properties but it also scales with the beam size as the beam propagates in TEM [18], being typically a few tens of nanometers.

## 5. Principle of Gabor Holography

**Figure 7:**
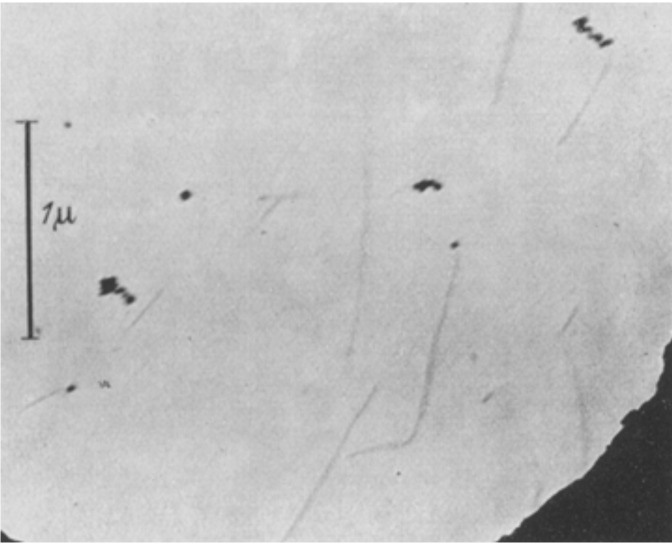
Images of the tobacco mosaic virus obtained by Gustav Kausche, Edgar Pfankuch, and Helmut Ruska in 1939, using an electron microscope. Reprinted from [22] by permission from Springer Nature, copyright 1939.

The first electron microscope was built by Ernst Ruska and Max Knoll between 1931 and 1933 [19]. The very short wavelengths of electrons gave rise to the hope that these microscopes could be used to visualize very small objects such as viruses. Although biologists had already identified the function and activity of viruses before the era of electron microscopy [20,21], their geometrical shapes remained a mystery, and with the invention of the electron microscope, it become possible to visualize these shapes. The first images of viruses were obtained by Gustav Kausche, Edgar Pfankuch, and Helmut Ruska in 1939 using an electron microscope; they imaged a tobacco mosaic virus (TMV) and identified its simple geometrical shape, a rod 18 nm in diameter and 300 nm in length [22], as shown in Figure 7. TMV has a remarkable history of “firsts”, since it was also the first virus to be discovered and named [21].

However, these electron microscope images of TMVs (also shown in Figure 7) had low resolution and quality. The question therefore arose as to why the resolution of the image was so low, despite the very short wavelength of the electrons used, which was much smaller than the typical interatomic distance of 1.5–2 Å, and this issue occupied many scientists at the time. In 1936, Otto Scherzer published his research on the subject (now known as the Scherzer theorem), in which he studied the properties of an imaging system using electromagnetic lenses. Scherzer demonstrated that because of the symmetry of the electromagnetic lenses, static electromagnetic fields and the absence of space charges (properties of a typical electromagnetic lens system), aberrations (mainly chromatic and spherical) will always be present and will degrade the resolution, thus preventing atomic resolution images [23]. Scientists therefore started to search for solutions to the aberrations problem. Eventually, aberration-corrected transmission electron microscopes (ACTEM) were developed in 2010, which delivered images with atomic resolution. However, there was also another outstanding solution to the aberrations problem, which resulted in a completely new technique—holography.

**Figure 8:**
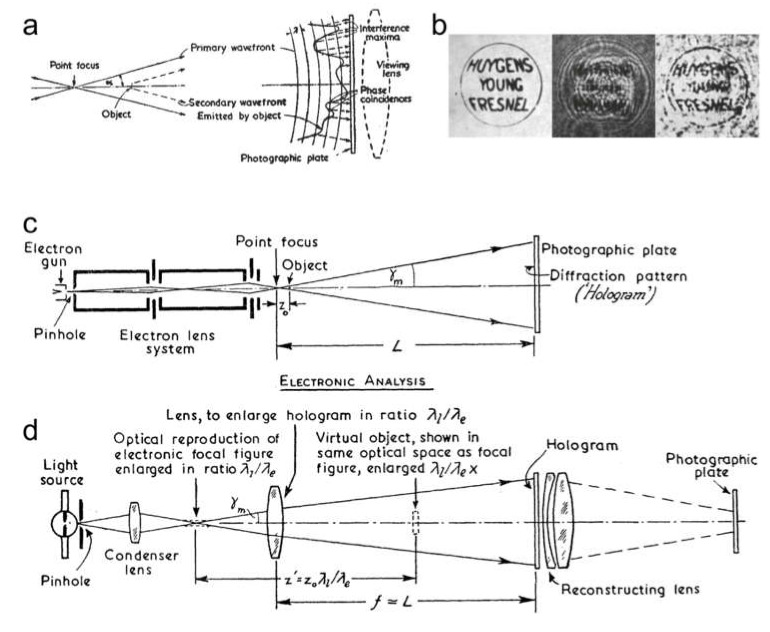
Principle of holography, as illustrated by Dennis Gabor [25,26]. (**a**,**c**) schematic of realization of holography in a transmission electron microscope. (**b**) Sample (left) with three words written on a transparent film, its hologram (middle) recorded on photographic film, and the reconstructed hologram (right) as a result of optical holography experiments involving recording and reconstruction of holograms, as shown in (**d**). (**a**,**b**) Reprinted from [25] by permission from Springer Nature, copyright 1948.

In 1947, Dennis Gabor patented a novel imaging technique which he named “holography” [24,25,26]. Gabor’s ingenious idea for solving the aberrations problem in an electron microscope was to remove all the lenses between the sample and the detector. He argued that since the electron wave that was partially scattered by the sample (object wave) would interfere with the unscattered (reference) wave, the resulting interference pattern formed in the detector plane would contain the complete information about the object wave, and the entire object distribution could therefore be reconstructed. Although Gabor envisioned this new type of microscopy being applied in an electron microscope (Figure 8a,c), he proved this principle using an optical experiment (Figure 8b,d) [25,26]. This form of holography is called Gabor-type or in-line holography, since the object and the reference wave share the same optical axis.

## 6. Point Projection Microscopy (PPM)

**Figure 9:**
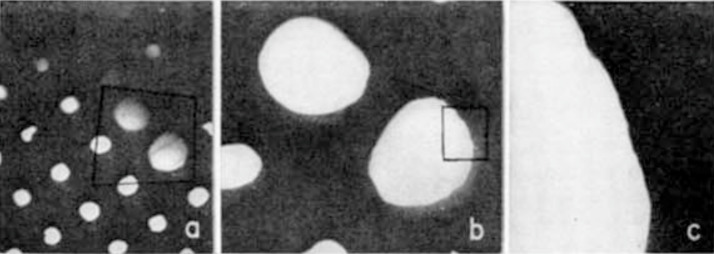
Images of a copper grid obtained by Morton and Ramberg using point projection microscopy (PPM) with electrons, at magnifications of (**a**) 200, (**b**) 600, and (**c**) 3000 times. Figure reprinted from [27], copyright (1939) by the American Physical Society.

A frequently used experimental scheme that is similar to in-line holography is called point projection microscopy (PPM). The reason for these different names for two almost identical experimental arrangements is as follows. In 1939, George A. Morton and Edward G. Ramberg published a half-page article entitled “Point projector electron microscope,” in which they described a novel type of electron microscopy [27]. Their technique employed “an etched tungsten or molybdenum point” cathode as an electron source, a specimen that was partially transparent, and no lenses between the sample and the detector. The image formed on the detector was a magnified image of the sample, where the magnification was determined by the ratio between the source-to-detector and the source-to-sample distances. They produced experimental images of a copper grid, as shown in Figure 9. In their experiments, Morton and Ramberg achieved a magnification of up to 8000 times, but did not publish their images at this magnification because, as they explained, the quality of the images was degraded because of insufficient mechanical steadiness. However, it is possible that this reduction in the quality of the images (that is, the degraded sharpness of the edge) was in fact caused by the diffraction and interference effects which arise at shorter source-to-sample distances. Thus, Morton and Ramberg may have observed the first holograms already in 1939.

**Figure 10:**
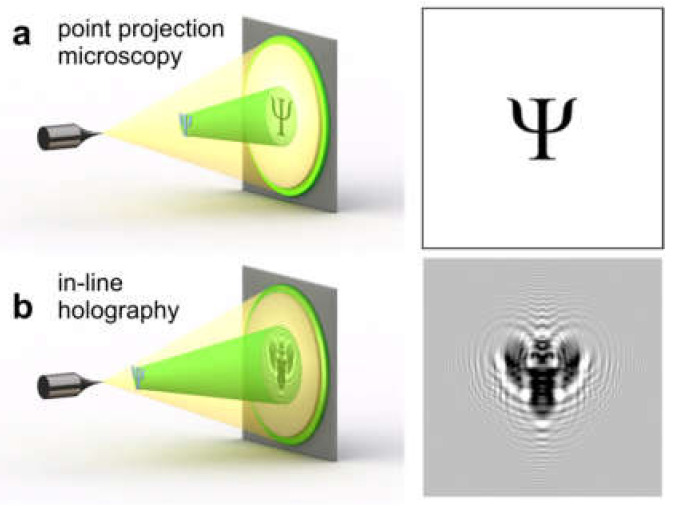
Point projection microscopy (PPM) and in-line holography. (**a**) Experimental arrangement for PPM and the resulting image. (**b**) Experimental arrangement for in-line holography and the resulting image.

The main difference between PPM and in-line holography is that in the former, the resulting image is a projection image rather than an interference pattern (Figure 10a), while in the latter, the resulting image is an interference pattern formed by interference between the scattered and non-scattered waves (Figure 10b). Thus, the same experimental setup can be utilized in two regimes, since at shorter source-to-sample distances, an interference pattern emerges and a point projection image turns into a hologram. This is why this experimental arrangement is referred to by some researchers as PPM [28,29,30,31,32,33,34,35,36], and by others as in-line holography [37,38]. Another point which should be mentioned is that in the in-line holography proposed by Gabor, the point source is a focused spot, rather than a physical source as in PPM. 

## 7. Off-Axis Holography

### 7.1. The Electron Biprism

**Figure 11:**
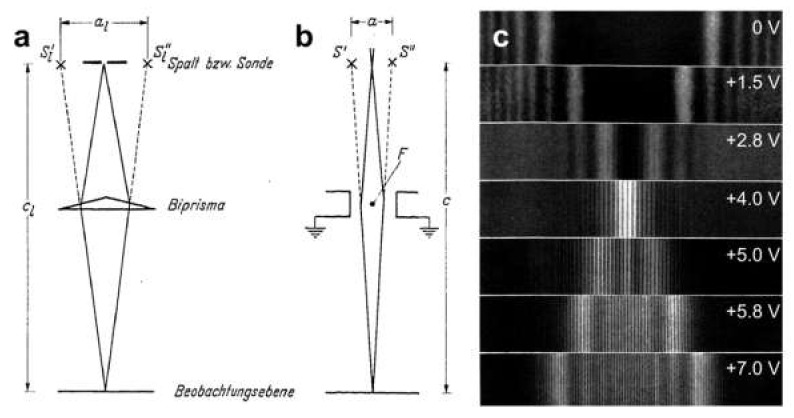
A biprism in an electron microscope. Ray diagrams for (**a**) an optical prism and (**b**) an electron biprism in an electron microscope. (**c**) Experimentally recorded electron biprism interference patterns with different potentials applied to the biprism, using a wire 2 μm in diameter. Reprinted from [39] by permission from Springer Nature, copyright 1956.

In 1956, Möllenstedt and Düker invented a method for splitting electron beams by placing a positively charged wire within the electron wave, orthogonal to the propagation of the wavefront. In this scheme, electrons passing the positively charged wire are deflected toward the wire, and thus the electron wave is split into two overlapping wavefronts, creating an interference pattern of equidistant fringes, as illustrated in Figure 11. The electron biprism thus acts in an analogous way to an optical prism (hence the term “biprism”) [39]. 

### 7.2. Measuring Potentials Using Off-Axis Holography

#### 7.2.1. Electrostatic Potential

**Figure 12:**
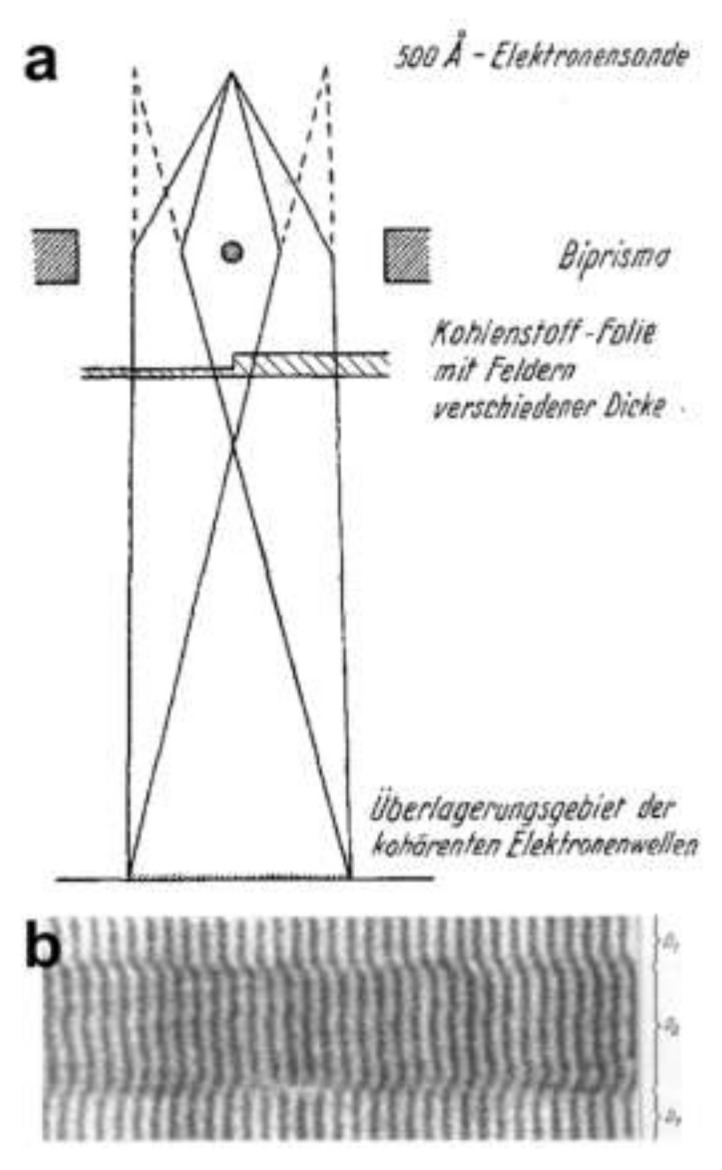
Off-axis electron holography. (**a**) Experimental arrangement. (**b**) Off-axis electron hologram exhibiting the shift in the interference pattern caused by the different thicknesses of the sample, and therefore by the differences in potential and the additional phase shift. Reprinted from [40] by permission from Springer Nature, copyright 1957.

In 1957, one year later after demonstrating the principle of the electron biprism, Möllenstedt and Keller placed a sample into the one of the two split electron beams and measured the resulting interference pattern, thus creating the first off-axis electron hologram [40]. Their experimental arrangement is shown in Figure 12a. Based on the acquired interference pattern, they were able to measure the electrostatic potential of a sample as follows. The sample consisted of strips of carbon film of different thicknesses, 40 and 160 Å, and the thickness was measured by optical absorption. The accelerating voltage was U=54.4 kV. The phase shift caused by different potentials was evaluated as $\Delta \varphi =2\pi \left(\frac{D}{\lambda_{m}}-\frac{D}{\lambda_{v}}\right)=-2\pi D\frac{\Delta \lambda }{\lambda^{2}}$ where $\lambda_{v}$ is the wavelength in a vacuum, $\lambda_{m}$ is the wavelength in the material, D is the difference in thickness, and here D=120 Å. $\lambda =\sqrt{\frac{150}{U}}$ (λ in Å, U in volts), thus giving $\Delta \lambda =-\frac{1}{2}\sqrt{\frac{150}{U^{3}}}\Delta U$. From the bending of the fringes in the interference pattern, which correspond to the regions of different sample thickness (as shown Figure 12b), Möllenstedt and Keller evaluated the phase shift to be about Δφ=π±15%, and calculated the potential as $\Delta U=\frac{300\Delta \varphi }{2\pi D\lambda }=\left(24\pm 5\right)$ V [40]. Since this first experiment, the measurement of electrostatic potentials has been one of the main applications of electron off-axis holography [41,42,43]. Recently, high-energy off-axis electron holography has been applied for imaging individual charges and the electrostatic charge density distributions with a precision of better than a single elementary charge [44,45].

#### 7.2.2. Magnetic Potential

The possibility of measuring the magnetic potential was proposed shortly after the above demonstration of measuring the electric potential using off-axis electron holography. In 1959, Yakir Aharonov and David Bohm published a theoretical paper in which they described a quantum mechanical phenomenon whereby an electrically charged particle will be affected by an electromagnetic potential, despite being confined to a region in which both the magnetic field B and electric field E are zero [46]. This phenomenon is known now as the Aharonov-Bohm (AB) effect, and since it cannot be explained in the frame of classical electrodynamics, it is a truly quantum phenomenon. In their paper, Aharonov and Bohm even provided a sketch of an experimental scheme based on electron interference, which was an arrangement very similar to off-axis holography. In 1960, the corresponding experiment was conducted by Chambers [47], who demonstrated a shift in an electron interference pattern caused by an enclosed magnetic flux, thus proving the AB effect and the quantum nature of the electronic interaction with the magnetic potential. Nowadays, electron off-axis holography is routinely applied in measuring the magnetic properties of material science and biological samples in transmission electron microscopes operating with keV energy electrons [10,48,49,50,51,52].

### 7.3. Reconstruction of an Off-Axis Hologram

Superimposing a tilted reference wave $U_{R}$ and an object wave $U_{O}$, where

(41) $$\begin{array}{l} U_{R}\left(\vec{r}\right)=\exp\left(i{\vec{q}}_{R}\vec{r}\right),\\ U_{O}\left(\vec{r}\right)=A_{O}\left(\vec{r}\right)\exp\left[i\varphi_{O}\left(\vec{r}\right)\right], \end{array}$$

yields an interference pattern with

(42) $$I\left(\vec{r}\right)=1+{\left|A_{O}\left(\vec{r}\right)\right|}^{2}+2A_{O}\left(\vec{r}\right)\cos\left[{\vec{q}}_{R}\vec{r}+\varphi_{O}\left(\vec{r}\right)\right],$$

where the tilt of the reference wave is specified by the two-dimensional reciprocal space vector ${\vec{q}}_{R}$, $A_{O}\left(\vec{r}\right)$ and $\varphi_{O}\left(\vec{r}\right)$ refer to amplitude and phase, respectively. From Equation (42), it can be shown that the FT of a hologram can be written in the form

(43) $$\mathrm{FT}\left(I\right)=\delta \left(\vec{q}\right)+\mathrm{FT}\left({\left|A_{O}\right|}^{2}\right)+\delta \left(\vec{q}+{\vec{q}}_{R}\right)\mathrm{FT}\left[A_{O}\exp\left(-i\varphi_{O}\right)\right]+\delta \left(\vec{q}-{\vec{q}}_{R}\right)\mathrm{FT}\left[A_{O}\exp\left(i\varphi_{O}\right)\right].$$

**Figure 13:**
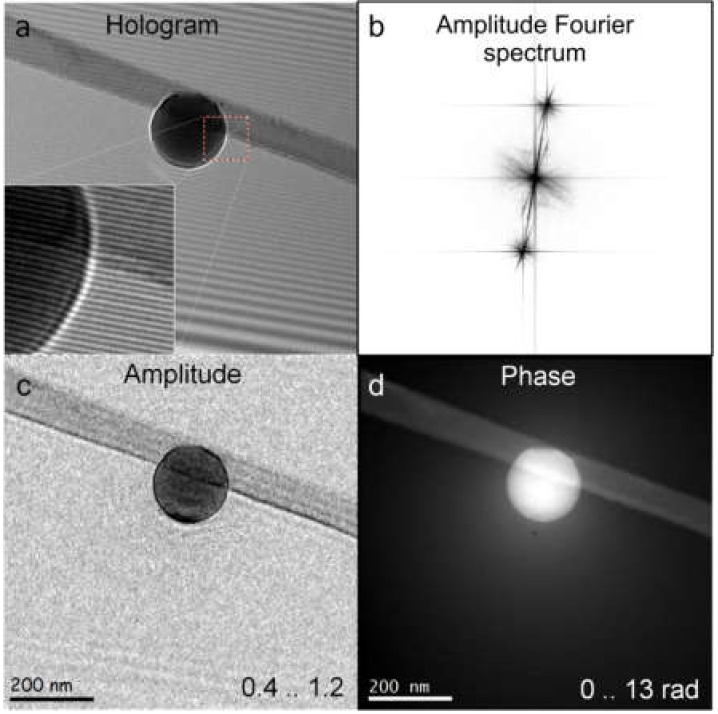
Electron off-axis hologram of a latex sphere and its reconstruction. (**a**) Off-axis hologram of a latex sphere recorded at 200 keV, with Fresnel fringes from the biprism filament edge readily visible. (**b**) Amplitude of the Fourier spectrum of the hologram shown in (**a**). (**c**) Reconstructed amplitude. (**d**) Unwrapped reconstructed phase, with phase values between 0 and 13 rad. Reprinted from [53], with permission from Elsevier.

The resulting 2D complex-valued Fourier spectrum consists of the autocorrelation (central band) and two mutually conjugated sidebands centered at the carrier frequencies (${\vec{q}}_{R}$ and $-{\vec{q}}_{R}$), as shown in Figure 13b.

The numerical reconstruction of an off-axis hologram (recorded with light, electrons, or any other radiation) consists of the following steps, and an example is shown in Figure 13. (i) A 2D FT of the hologram (Figure 13a) is calculated. (ii) In the resulting 2D spectrum (Figure 13b), one of the two sidebands is selected by applying a low-pass filter centered on the chosen sideband, setting the central band and the other sideband to zero. (iii) The selected sideband is shifted to the center of the spectrum. (iv) The resulting complex-valued spectrum is then inverse Fourier transformed back to the real space. (v) The 2D amplitude (given by $A_{O}\left(\vec{r}\right)$) and phase (given by $\varphi_{O}\left(\vec{r}\right)$) distributions are extracted from the obtained distribution, as shown in Figure 13c,d.

### 7.4. Low-Energy Electron Off-Axis Holography

**Figure 14:**
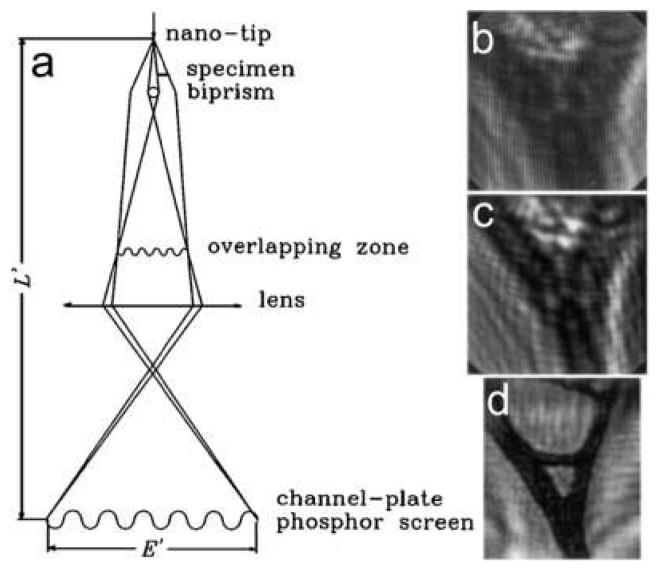
Low-energy electron off-axis holography. (**a**) Schematic arrangement of the low-energy holographic electron microscope with a biprism. (**b**–**d**) Imaging of a perforated carbon foil: (**b**) off-axis hologram, (**c**) in-line hologram without biprism, and (**d**) amplitude reconstructed from (**b**). The field of view is 217 nm. Figure reprinted from [56], copyright (1996) by the American Physical Society.

Off-axis holography in a low-energy electron microscope has been demonstrated by Roger Morin and colleagues, and has been reported in several publications [54,55,56,57,58]. A schematic of the experimental arrangement is similar to the PPM or Gabor in-line holography, shown in Figure 14a. The sample is placed into a divergent electron wave, and the biprism is placed between the sample and the detector. An electrostatic lens is used to magnify the image of the obtained interference pattern. Since PPM and Gabor in-line holography do not use any lenses, the image of the sample is always a defocused (due to Fresnel diffraction) image of the sample. Morin and colleagues reported a series of experiments imaging carbon foil (an example is shown in Figure 14b–d) [54,55,56,57,58]; they also imaged a sharp magnetic (Ni) tip above and below the Curie temperature and observed the phase shift related to the magnetic flux [58].

### 7.5. Further Reading about Off-Axis Holography

For further reading about off-axis electron holography, numerous papers by Hannes Lichte and his colleagues can be recommended as tutorials [13,59,60,61]. The applications of off-axis electron holography in materials science are discussed in references [51,62], and for biological samples in references [63,64]. The performance limits of off-axis holography are discussed by Lichte in reference [65].

## 8. In-Line Holography

### 8.1. In-Line Holography in TEM

#### 8.1.1. Defocused, Over-Focused, and Under-Focused Imaging

In-line holography is easily realized in TEM by simply defocusing the image of the sample. In this case, the interference between the unscattered and scattered waves forms the in-line hologram. A particularly interesting case is that of phase objects. Most biological macromolecules such as proteins are composed of atoms (C, H, O, N) which are relatively weak scatterers, so that the entire macromolecule is a weak phase object and creates no contrast when imaged in focus. The phase object only causes significant contrast when imaged in defocus.

**Figure 15:**
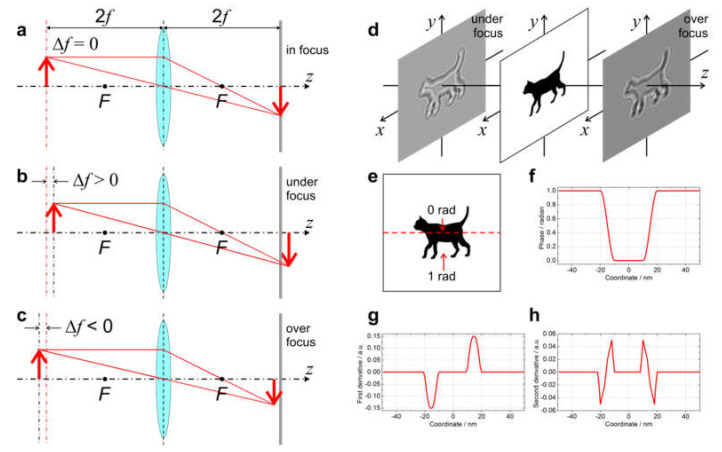
Defocused imaging in TEM. (**a**–**c**) Ray diagrams of a lens system when imaging (**a**) in focus, (**b**) under focus, and (**c**) over focus. The detector is at the same position in all three cases. The position of the object is shifted along the optical axis in (**b**) by Δf>0 and in (**c**) by Δf<0, and as a result, the image on the detector appears under-focused in (**b**) and over-focused in (**c**). (**d**) Under- and over-focused images of a pure phase object, a cat-shaped hole in a carbon film with the phase distribution shown in (**e**). (**f**–**h**) show profiles through the middle of the 2D distribution of (**f**) the sample, (**g**) its first derivative, and (**h**) its second derivative.

A defocused image can be obtained in the over- or under-focused regime, as illustrated in Figure 15a–c. This change in contrast can be explained using the transport of intensity (TIE) equation [66]:

(44) $$\frac{\partial I\left(x,y,z\right)}{\partial z}+\nabla_{x,y}\left[I\left(x,y,z\right)\frac{\nabla_{x,y}\varphi \left(x,y\right)}{k}\right]=0$$

where $I\left(x,y,z\right)$ is the intensity and $\varphi \left(x,y\right)$ is the phase distribution at a plane *z*. For a phase object imaged in focus, the wavefront is given by $U\left(x,y,z\right)=\exp\left[i\varphi \left(x,y\right)\right]$, the intensity $I\left(x,y,z\right)=1$ and Equation (44) becomes

(45) $$\frac{\partial I\left(x,y,z\right)}{\partial z}+\frac{1}{k}\nabla_{x,y}^{2}\varphi \left(x,y\right)=0.$$

By replacing the differential with a numerical differentiation, as $\frac{\partial I\left(x,y,z\right)}{\partial z}\approx \frac{I\left(x,y,z+\Delta z\right)-I\left(x,y,z\right)}{\Delta z}$, we can rewrite the TIE Equation (45) as

(46) $$I\left(x,y,z+\Delta z\right)=1-\frac{\lambda \Delta z}{2\pi }\nabla_{x,y}^{2}\varphi \left(x,y\right).$$

In Equation (46), the intensity distribution is proportional to the second derivative of the phase distribution. 

A simulated example is shown in Figure 15d–h. Here, the test object is a hole in a thin carbon film that causes a phase shift of 1 radian. From experimental observations, it is known that the under- and over-focused images of such a sample have clear signatures: the edge of the hole exhibits a bright fringe in the under-focused image, and a dark fringe in the over-focused one. The second derivative of the phase distribution is shown in Figure 15h. For the under-focused image, Δf>0 and Δz<0, and according to Equation (46), $I\left(x,y,z+\Delta z\right)\propto \nabla_{x,y}^{2}\varphi \left(x,y\right),$ giving a bright fringe at the edge of the hole. For the over-focused image, Δf<0 and Δz>0, and according to Equation (46), $I\left(x,y,z-\Delta z\right)\propto -\nabla_{x,y}^{2}\varphi \left(x,y\right),$ meaning that a dark fringe is seen at the edge of the hole.

#### 8.1.2. Focal (Defocus) Series

**Figure 16:**
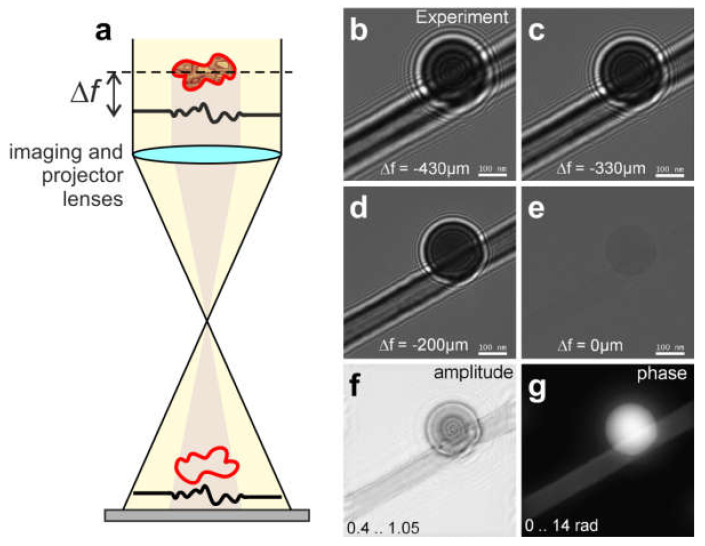
Defocus series in transmission electron microscope (TEM). (**a**) Drawing of a holographic in-line scheme, where the red (yellow) color represents the object (reference) wave and Δf is the defocus distance. (**b**–**e**) Experimental in-line holograms of a latex sphere recorded at different values of defocus. (**f**) Amplitude and (**g**) phase of the object wave, reconstructed using an iterative flux-preserving focal series reconstruction algorithm [71]. Reprinted from [53], with permission from Elsevier.

In 1986, Schiske proposed the possibility of full wavefront reconstruction from a sequence of intensity measurements acquired at different defocus distances in an electron microscope [67]. In 1992, Coene et al. demonstrated the unambiguous high-resolution reconstruction of samples obtained from a focal series acquired in a TEM, and this has become a practical tool for image analysis in high-resolution transmission electron microscopy (HRTEM) [68]. HRTEM images of material science samples (and particularly crystals) often display misleading contrast; for example, bright spots can be mistaken for atoms but in reality are the spaces between atoms. Strictly speaking, HRTEM images cannot be interpreted alone, and corresponding simulations must be performed to match the experimental images. For an unambiguous determination of the structure, focal image series can be acquired and reconstructed using numerical procedures, thus recovering the complex-valued exit wave at atomic resolution [69]. Focal series of images can be reconstructed by employing the TIE [66], as has been demonstrated for light optical [70] and electron holograms [71] or by iterative phase retrieval methods. A sequence of in-line electron holograms acquired at different defocus distances and their reconstruction, obtained using the flux-preserving iterative reconstruction algorithm described in [71], are shown in Figure 16.

#### 8.1.3. Single In-Line Hologram and Its Reconstruction

**Figure 17:**
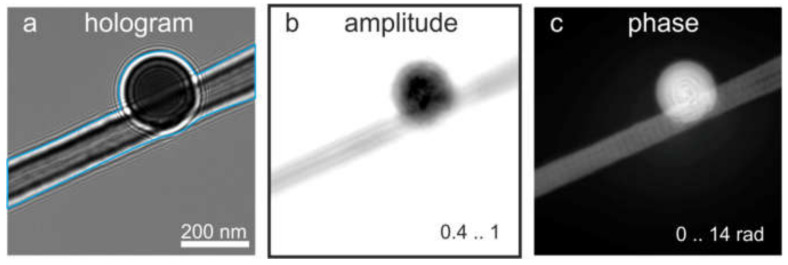
In-line hologram of a latex sphere and its reconstruction. (**a**) In-line electron hologram of the latex sphere recorded at the defocus 180 μm, with 200 keV electrons in a TEM. The blue lines mark the area outside of which the transmission was set to 1 during the iterative reconstruction (support). (**b**,**c**) show the retrieved amplitude and phase distributions, respectively. Reprinted from [53], with permission from Elsevier.

The object distribution can be also reconstructed from a single in-line hologram (defocus image) by applying iterative reconstruction, as explained in detail in the literature [53,72,73,74,75,76]. We provide only a brief summary of the reconstruction steps here. Before reconstruction, the hologram is divided by the background image, which is recorded under exactly the same experimental conditions as the hologram, only without the object. Alternatively, the background image can be created numerically by simple low-pass filtering of the hologram of the object. The hologram divided by the background image is the normalized hologram. The distribution of this normalized hologram does not depend on the parameters such as the intensity of the reference wave, and is described mathematically by the holographic equation with a reference wave of amplitude one. The normalized hologram can be reconstructed as described elsewhere [77], and quantitatively correct absorption and phase distributions of the sample can be extracted [78]. Next, an iterative reconstruction routine is applied based on the Gerchberg-Saxton algorithm [79], in which the wavefront is propagated back and forth between the two planes (i.e. the hologram and object planes), and constraints are applied in each plane. In the hologram plane, the updated amplitude is replaced with the square root of the measured intensity, while in the object plane, support constraint [80,81], positive absorption constraint [72], and/or real and positive constraints can be applied to the reconstructed object distribution. An example of a latex sphere reconstructed from a single in-line electron hologram (obtained by defocused imaging in a TEM) with positive absorption and finite support constraints is shown in Figure 17. Here, the sample exhibits absorption and a significant phase shift [53].

### 8.2. Low-Energy Electron Holography

#### 8.2.1. Experimental Arrangement

**Figure 18:**
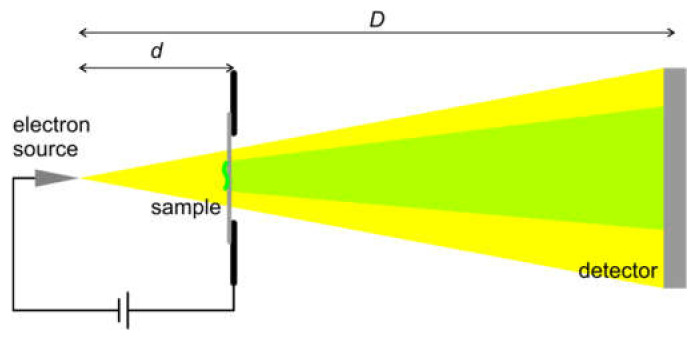
Experimental arrangement for in-line holography with low-energy electrons.

An experimental arrangement for in-line holography with low-energy electrons [37,82] is sketched in Figure 18. Electrons are extracted by field emission from a sharp tungsten W (111) tip, with energy 30–250 eV. A sample is placed in front of the tip at a distance d from the source, where d ranges from tens of nanometers to a few microns. The in-line hologram formed by the interference between the scattered and non-scattered wave is acquired by a detector positioned at a distance D from the source, D is typically 5–20 cm. The magnification of the microscope is given by the ratio D/d. The technical details of low-energy holographic microscopes are provided in references [37,83,84].

#### 8.2.2. Reconstruction of In-Line Holograms

Algorithms for the simulation and reconstruction of in-line holograms are provided and explained in detail in reference [77]. Here, we discuss the main conclusions of the theory of formation and reconstruction of in-line holograms. 

Plane waves. In-line holography is often realized with plane waves. In this case, the incident wavefront is a plane wave, and the interference pattern (hologram) is formed at some not too far distance from the sample. The distribution of the interference pattern changes with the distance from the sample. The complex-valued wavefront at the detector is given by the Fresnel diffraction integral, and in the paraxial approximation can be calculated as a convolution:

(47) $$\begin{array}{l} U_{\mathrm{detector}}\left(X,Y\right)=-\frac{i}{\lambda }\iint_{S}t\left(x,y\right)\exp\left\{\frac{i\pi }{\lambda z}\left[{\left(x-X\right)}^{2}+{\left(y-Y\right)}^{2}\right]\right\}dxdy\\ \propto t\left(X,Y\right)⊗\exp\left[\frac{i\pi }{\lambda z}\left(X^{2}+Y^{2}\right)\right] \end{array}$$

where $t\left(x,y\right)$ is the transmission function of the sample, $\left(x,y\right)$ are the coordinates in the sample plane, $\left(X,Y\right)$ are the coordinates in the detector plane, z is the distance between the sample and the detector, and ⊗ denotes convolution.

Spherical waves. The original in-line Gabor-type holography employed a divergent incident wavefront. In this arrangement, even though the resulting interference pattern (hologram) is acquired in the far field, the distribution of the diffracted wave is described by Fresnel diffraction. The interference pattern has the same appearance at any distant detecting plane, and moving the detecting plane away from the sample will result only in an increased magnification of the interference pattern. However, changing the distance between the source and the sample will change the distribution of the interference pattern (hologram); that is, it will have the same effect as changing the sample-to-detector distance in in-line holography with plane waves. 

The complex-valued wavefront at the detector plane is given by the Fresnel diffraction integral, and in the paraxial approximation can be calculated as a convolution:

(48) $$\begin{array}{l} U_{\mathrm{detector}}\left(X,Y\right)=-\frac{i}{\lambda }\iint_{S}\exp\left[\frac{i\pi }{\lambda z_{1}}\left(x^{2}+y^{2}\right)\right]t\left(x,y\right)\;\exp\left\{\frac{i\pi }{\lambda \left(z_{2}-z_{1}\right)}\left[{\left(x-X\right)}^{2}+{\left(y-Y\right)}^{2}\right]\right\}dxdy\\ \propto \iint_{S}t\left(x,y\right)\exp\left\{\frac{i\pi }{\lambda z_{1}}\left[{\left(x-\frac{z_{1}}{z_{2}}X\right)}^{2}+{\left(y-\frac{z_{1}}{z_{2}}Y\right)}^{2}\right]\right\}dxdy, \end{array}$$

where $z_{1}$ is the distance between the source and the sample, and $z_{2}$ is the distance between the source and the detector. By introducing the scaled coordinates $X^{\prime }=\frac{z_{1}}{z_{2}}X=\frac{X}{M}$ and $Y^{\prime }=\frac{z_{1}}{z_{2}}Y=\frac{Y}{M}$, where $M=\frac{z_{2}}{z_{1}}$ is the magnification factor, Equation (48) can be written as:

(49) $$U_{\mathrm{detector}}\left(X^{\prime },Y^{\prime }\right)\propto o\left(X^{\prime },Y^{\prime }\right)⊗\exp\left[\frac{i\pi }{\lambda z_{1}}\left(X^{\prime 2}+Y^{\prime 2}\right)\right]$$

which implies that a hologram recorded with a spherical wave with source-to-sample distance $z_{1}$ can be treated as a hologram recorded with a plane wave with sample-to-detector distance $z_{1}$, and the coordinates are scaled by a magnification factor M.

**Figure 19:**
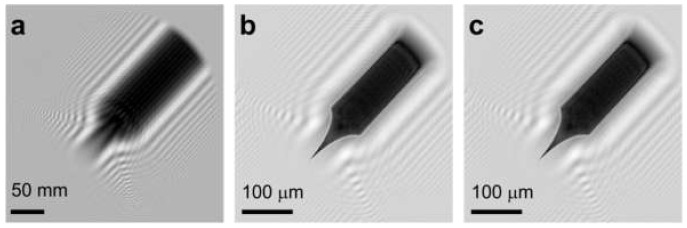
Optical hologram of a tungsten tip and its reconstruction. (**a**) Hologram recorded with 532 nm laser light in an in-line Gabor scheme with spherical waves, with a source-to-sample distance of 1.4 mm, and a source-to-screen distance of 1060 mm. (**b**) Amplitude of the object distribution reconstructed from the hologram shown in (**a**) using the reconstruction algorithm for spherical waves, where the size of the reconstructed area is 429 × 429 μm^2^. (**c**) Amplitude of the object distribution from the hologram shown in (**a**) using the reconstruction algorithm for plane waves, assuming a hologram size of 429 × 429 μm^2^ and a sample-to-hologram distance of 1.4 mm. Adapted from [77].

For a thin sample that can be approximated by a 2D distribution in one plane, a hologram acquired with a spherical wave can be reconstructed as if it had been obtained with a plane wave, as illustrated in Figure 19. The following relation holds [77]:

(50) $$\frac{\lambda z}{S_{\mathrm{plane}}^{2}}=\frac{\lambda z_{2}^{2}}{z_{1}S_{\mathrm{spherical}}^{2}}$$

where *S*_plane_ × *S*_plane_ and *S*_spherical_ × *S*_spherical_ are the sizes of the hologram recorded with plane and spherical waves, respectively.

#### 8.2.3. Imaging Biological Samples and Individual Macromolecules

Low-energy electrons with kinetic energies in the range 30–250 eV have the advantage of causing no significant radiation damage to biological molecules; this has been exemplified by the continuous exposure of individual DNA molecules to low-energy electrons for 70 min, without a noticeable change in their in-line holograms at a resolution of 1 nm [85,86]. The number of electrons required to acquire a single 20 ms low-energy electron hologram at a resolution of 1 nm amounts to about 250 electrons per 1 Å^2^, which translates into a radiation dose of 4.58 × 10^11^ Gray. 

**Figure 20:**
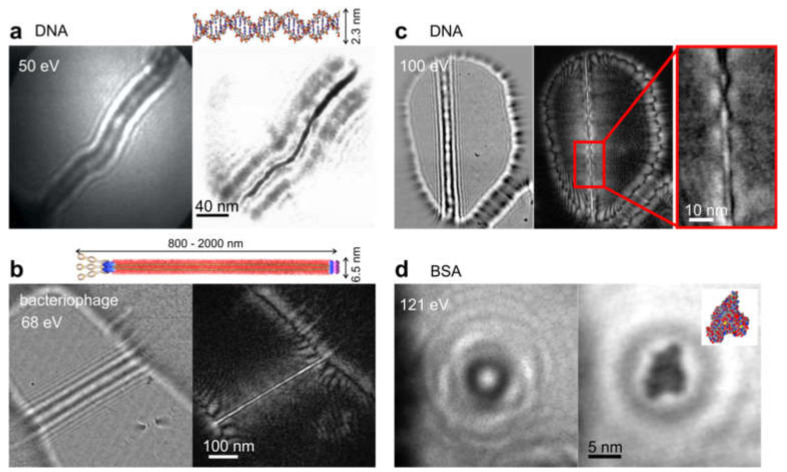
Low-energy in-line holography imaging of individual macromolecules, showing results obtained by Fink et al. University of Zurich. In each case, the left image shows experimental holograms and the right shows the corresponding reconstructions: (**a**) DNA molecules, copyright OSA 1997 [38], (**b**) bacteriophage molecule (reprinted by permission from Springer Nature [90], copyright 2011), (**c**) DNA molecule [87] (copyright Springer Nature 2013), and (**d**) bovine serum albumin (BSA) molecules [92].

**Figure 21:**
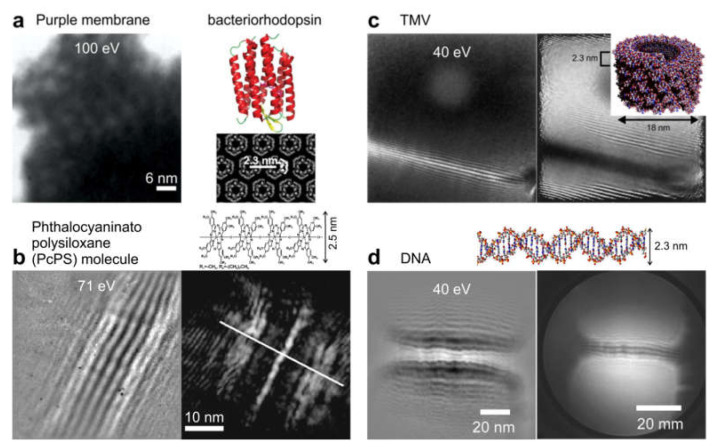
Low-energy in-line holography imaging of individual macromolecules. In each case, the left image shows experimental holograms, and the right shows the corresponding reconstructions. (**a**) Purple membrane (reprinted from [84], with permission from Elsevier). (**b**) Phthalocyaninato polysiloxane (PcPS) molecule (reprinted with permission from [28], copyright 1998, American Vacuum Society). (**c**) Tobacco mosaic virus (TMV) (reprinted from [88], with permission from Elsevier). (**d**) DNA molecules [30].

Low-energy electron in-line holography has been successfully applied to the imaging of various individual biological molecules, for example purple protein membrane [84], DNA molecules [30,38,86,87], phthalocyaninato polysiloxane molecule [28], TMV [88,89], a bacteriophage [90], ferritin [91], and individual proteins (bovine serum albumin, cytochrome C, and hemoglobin) [92]; some of these results are shown in Figure 20 and Figure 21.

#### 8.2.4. Imaging Electric Potentials

**Figure 22:**
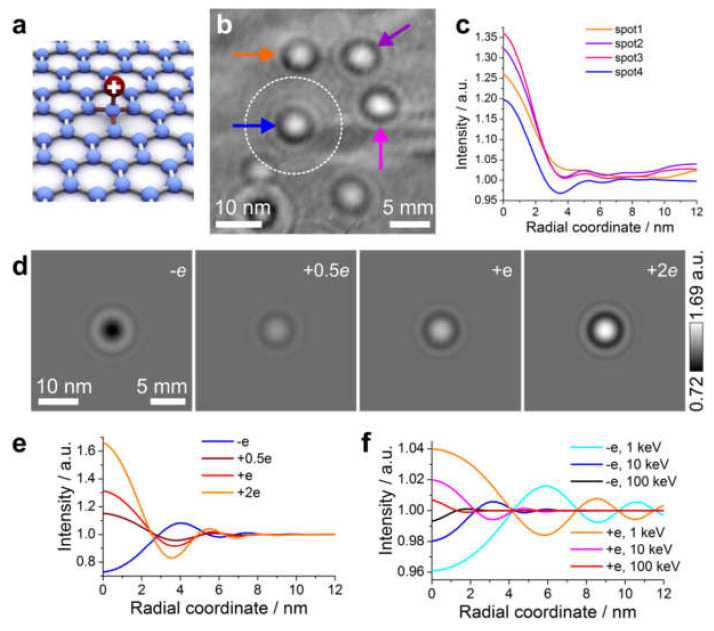
In-line electron holograms of charged adsorbates. (**a**) Schematic representation of a charged adsorbate on graphene. (**b**) Experimental hologram exhibiting bright spots; here, the electron energy is 30 eV, the source-to-sample distance is 82 nm and the source-to-screen distance is 47 mm. (**c**) Angular-averaged intensity profiles of the four bright spots marked in (**b**). (**d**) Simulated in-line holograms of a point charge, at four different values of charge, where the simulation parameters match those of the experimental hologram shown in (**b**). (**e**) Angular-averaged intensity profiles as a function of the radial coordinate, calculated from the simulated holograms shown in (**d**). (**f**) Angular-averaged intensity profiles as a function of the radial coordinate calculated from the simulated holograms at different high energies of probing electrons. The scale bars in (**b**,**d**) indicate the sizes in the object plane (left) and in the detector plane (right). Adapted with permission from [94], Copyright (2016) American Chemical Society.

Local electric potentials such as those created by individual charged adsorbates on graphene [93] can be visualized using low-energy electron in-line holography [94], with a sensitivity of a fraction of an elementary charge. Some results are shown in Figure 22. Low-energy electrons exhibit a sensitivity that is hundreds of times higher to local electric potentials than high-energy electrons. This can be explained intuitively based on the fact that an electron moving at a lower speed spends more time in the potential, and thus gets deflected more. An adsorbate with one elementary charge can cause about 30% and 1% contrast in an in-line hologram acquired with low- and high-energy (100 keV) electrons, respectively (Figure 22e,f). 

**Figure 23:**
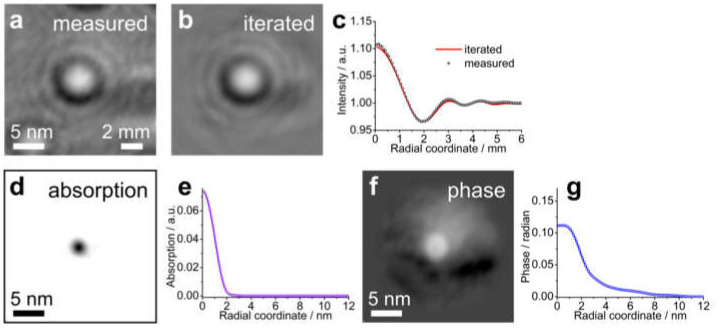
Iteratively reconstructed absorption and phase distribution of an individual charged impurity. (**a**) In-line hologram recorded with 30 eV electrons, exhibiting a bright spot. (**b**) Intensity distribution of the recovered wavefront obtained after 2000 iterations. (**c**) Angular-averaged radial profiles of the measured and iteratively recovered intensity distributions. (**d**,**f**) iteratively reconstructed absorption and phase distributions. (**e**,**g**) corresponding angular-averaged radial profiles. Reprinted from [74], with permission from Elsevier.

Iterative reconstruction of in-line holograms of individual charges provides the amplitude (associated with absorption) and phase (associated with the potential) distributions [74] (Figure 23). The reconstructed absorption distributions (Figure 23d,e) appear to be narrower than the reconstructed phase distributions (Figure 23f,g). This agrees well with the notion that the phase distribution (unlike the absorption distribution) does not reflect the actual size of the adsorbate, but instead reflects the potential distribution caused by the charge.

### 8.3. 3D Sample Reconstruction from Two or More In-Line Holograms

**Figure 24:**
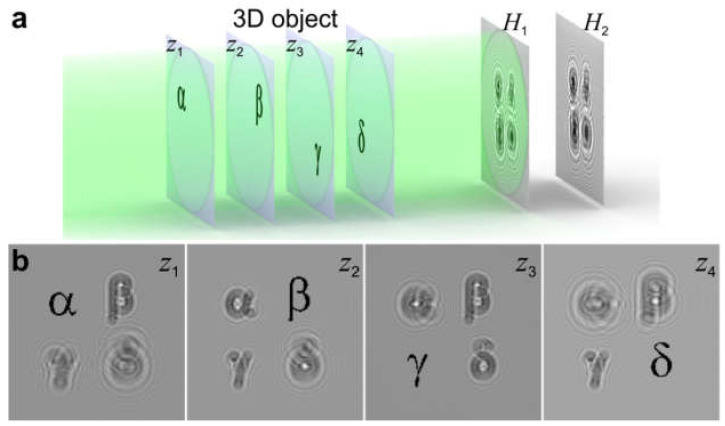
Reconstruction of 3D objects from two or more intensity measurements. (**a**) Experimental arrangement, in which the 3D sample is represented by a set of planes at different *z*-positions and two holograms are acquired at different distances from the sample, *H*_1_ and *H*_2_. (**b**) Reconstructed amplitude distributions at four planes within the 3D sample distribution. Adapted from [75].

For a thin sample that can be described by a 2D transmission function, a single-shot in-line hologram is sufficient to reconstruct the absorption and phase distributions of the sample. However, realistic physical objects always have some finite thickness, and therefore are rather 3D than 2D samples. In optical holography, the complete reconstruction of a wavefront from a sequence of intensity measurements using an iterative procedure has been demonstrated in series of studies between 2003 and 2006 [95,96,97,98,99]. It has been recently demonstrated that 3D samples, including 3D phase objects, can be reconstructed from two or more holograms recorded at different *z*-distances from the sample [75], as illustrated in Figure 24. This reconstruction is performed by applying iterative phase retrieval only between the planes in which the intensity distributions were measured (*H*_1_ and *H*_2_ in Figure 24a), i.e., without involving any planes within the sample and hence with no need for constraints on the sample. The recovered complex-valued wavefront is then propagated back to the sample planes, and the 3D distribution of the sample is reconstructed (Figure 24b). It has been shown that in principle, as few as two holograms are sufficient to recover the entire wavefront diffracted by a 3D sample, and there is no restriction on the thickness of the sample or on the number of diffraction events within the sample. The sample does not need to be sparse, and a reference wave is not required. This method can be applied to 3D samples, such as a 3D distribution of particles, thick biological samples, and so on, including phase objects. 

## 9. Coherent Diffraction Imaging (CDI) with Electrons 

### 9.1. CDI with High-Energy Electrons 

**Figure 25:**
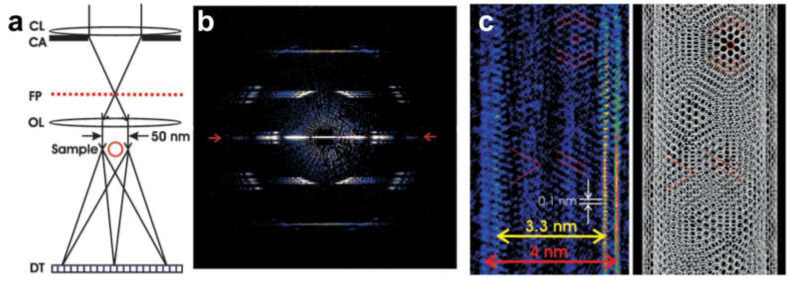
Coherent diffraction imaging of double-walled carbon nanotubes (DWCNTs) with high-energy electrons. (**a**) Schematic ray diagram of coherent nano-area electron diffraction. (**b**) Diffraction pattern of a DWCNT recorded with 200 keV electrons. (**c**) Section of the reconstructed DWCNT image at 1 Å resolution and (right) a structural model. Adapted from [115], reprinted with permission from AAAS.

CDI [100] is an imaging technique that is similar to diffraction in a crystal experiment, but involves imaging a single isolated object such as a macromolecule rather than a crystal [100,101,102,103,104,105,106,107,108,109,110]. In CDI, the structure of a sample is reconstructed from its diffraction pattern by applying an iterative phase retrieval algorithm [111,112,113]. To achieve this, the following requirements must be met: The object under study must be isolated; the size of the reconstructed field of view must exceed the size of the object by at least twice in each direction (oversampling condition) [113]; the incident wave must be a plane wave; and the imaging radiation must be coherent, although some attempts to employ partially coherent waves have been reported [114]. The power of CDI has been demonstrated by Zuo et al. who reconstructed the structure of a double-walled carbon nanotube (DWCNT) at atomic resolution from a diffraction pattern acquired using TEM with a nominal microscope point resolution of 2.2 Å for normal imaging at the Scherzer focus conditions [115], as shown in Figure 25. The results reported by Zuo et al. are often criticized, since the DWCNT had a finite size only in one dimension (*x*) and therefore the oversampling condition was not fulfilled for the other dimension (*y*), thus leading to an ambiguous reconstruction. However, it has recently been shown that for samples which can be described as a 1D chain of repeating units, or a 1D crystal, the average distribution of the repeating unit can be unambiguously reconstructed from the diffraction pattern of the sample, provided that the diffraction pattern is sufficiently oversampled [116]. Although the current study is limited to non-crystalline samples, we would like to add a notion that CDI of crystalline samples is highly challenging because of non-uniqueness of the reconstructed sample structure [117]. However, CDI can be successfully used for reconstruction of crystalline nano-particles when combined with other techniques [118,119].

### 9.2. CDI with Low-Energy Electrons 

**Figure 26:**
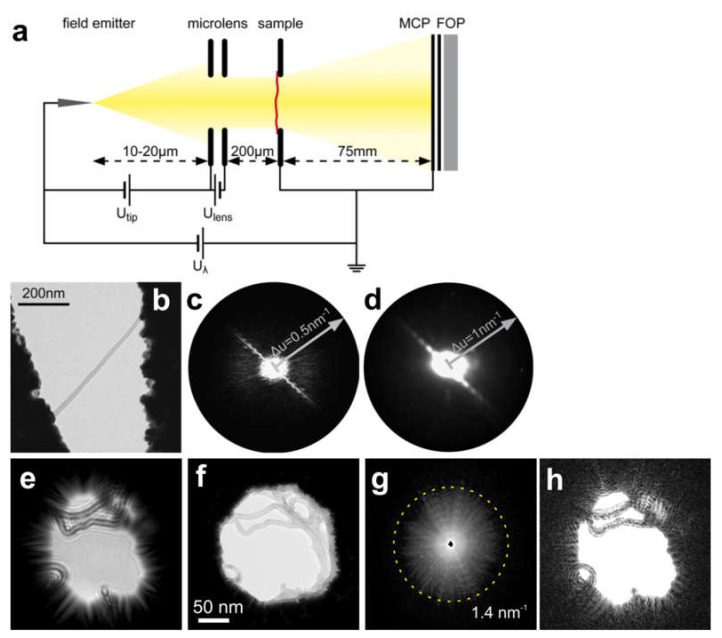
Coherent diffraction imaging (CDI) with low-energy electrons. (**a**) Experimental arrangement. (**b**–**d**) CDI of an individual single-walled carbon nanotube (SWCNT). (**b**) TEM image of the sample. (**c**) Fourier transform (FT) of the TEM image. (**d**) Diffraction pattern of SWCNTs recorded with 186 eV electrons. (**a**–**d**) reprinted from [121], with permission from Elsevier. (**e**–**h**) Holographic CDI (HCDI) reconstructions of a bundle of carbon nanotubes [122]. (**e**) In-line hologram recorded using electrons with kinetic energy 51 eV, source-to-sample distance 640 nm, and source-to-detector distance 68 mm. (**f**) TEM image recorded with 80 keV electrons. (**g**) Diffraction pattern recorded using electrons with kinetic energy 145 eV and source-to-detector distance 68 mm. The highest detected frequencies are indicated by the dashed circle, and the corresponding resolution is $R=\lambda /\left(2NA\right)=7$ Å. (**h**) Reconstructed amplitude distribution of the sample using HCDI. (**e**–**h**) Adapted from [122].

CDI with low-energy electrons has been demonstrated by Fink et al. in a dedicated low-energy electron microscope equipped with a microlens [120] to collimate the electron beam, as shown in Figure 26a. Low-energy electron diffraction patterns of individual macromolecules, such as carbon nanotubes [121,122] and graphene [9,123], were acquired. Diffraction patterns of individual stretched single-walled carbon nanotubes (SWCNTs) were acquired with 186 eV electrons at a resolution of 1.5 nm, as reported in reference [121], these results are shown in Figure 26b–d. Diffraction patterns of bundles of individual carbon nanotubes acquired with 145 eV electrons and reconstructed using a holographic CDI (HCDI) approach at a resolution of 0.7 nm were reported in reference [122], these are shown in Figure 26e–h. 

## 10. Discussion

### 10.1. In-Line Holography (Defocus Imaging) vs. CDI

**Figure 27:**
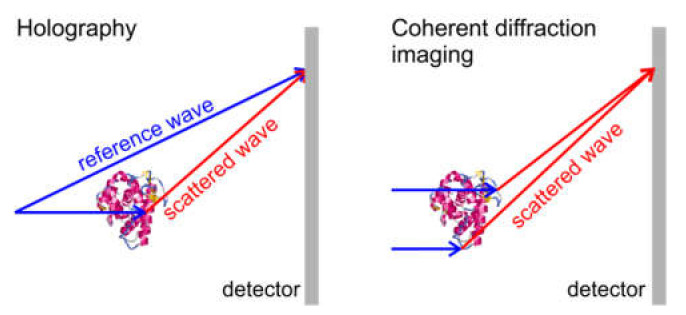
Schematic of (**a**) in-line holography and (**b**) coherent diffraction imaging (CDI).

In this subsection, we compare the two imaging schemes of in-line holography and CDI, both schemes are shown in Figure 27. Each scheme has certain advantages and disadvantages, as summarized in Table 1.

#### Radiation Dose

An important difference between in-line holography and CDI is the number of elastic scattering events (photons, electrons) required to obtain the sample reconstruction at a certain resolution. This difference is already evident from the principles of image formation in the two techniques: holography requires the object wave to be much weaker than the reference wave, while in CDI, only the object wave is measured, and it must therefore be sufficiently strong to be detected. In-line holography (or imaging in defocus) is often a suitable choice for imaging radiation-sensitive samples [124,125,126,127]. In the following, we provide a simple model for estimating and comparing the dose of radiation required in the two techniques to achieve a certain resolution. Here, we consider a phase object (phase 1 rad) of 10 nm in diameter probed with 200 keV electrons, although similar simulations and considerations can be done for electrons of different energy, or for photons. 

**Figure 28:**
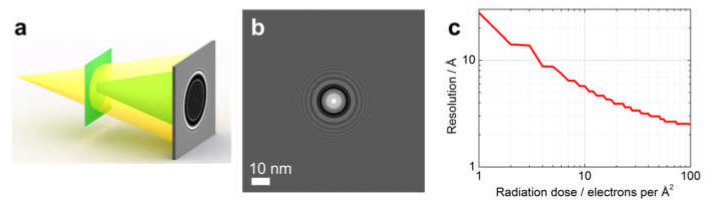
Radiation dose required to achieve a given resolution in in-line holography. (**a**) Experimental scheme for in-line holography. (**b**) Simulated in-line hologram of a round phase object of 10 nm in diameter. (**c**) Resolution as a function of radiation dose in in-line holography.

In-line holography (Figure 28): In-line electron holograms (Figure 28a) were simulated with the following parameters: illuminated area is 100 nm × 100 nm, electron energy is 200 keV, incident wave is a spherical wave, and source-to-sample distance is 10 μm (the same hologram distributions can be obtained with a plane wave at a defocus distance of 10 μm [77]). An example of the simulated hologram is shown in Figure 28b. The radiation dose was changed from 1 to 100 particles per Å^2^. For each radiation dose, a hologram was simulated and the hologram distribution was converged to integer numbers to mimic realistic detections of counts per pixel (cpp). The position of the highest detected interference fringes was then extracted, which defined the effective size of the hologram and the numerical aperture (*NA*). The resolution was calculated as $R=\lambda /\left(2NA\right)$ [122,128]. The resulting plot is shown in Figure 28c.

**Figure 29:**
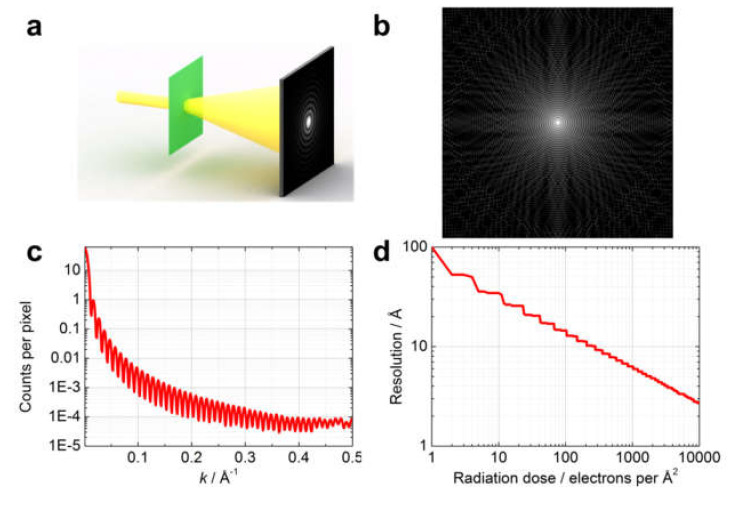
Radiation dose required to achieve a given resolution in CDI. (**a**) Experimental arrangement for CDI. (**b**) Simulated diffraction pattern of a round phase object of 10 nm in diameter. (**c**) Angular-averaged radial profile of a diffraction pattern simulated at a dose of 1 electron per Å^2^. (**d**) Resolution as a function of radiation dose in CDI.

CDI (Figure 29): Diffraction patterns (Figure 29a) were simulated at radiation doses ranging from 1 to 10,000 electrons per Å^2^. An example of simulated diffraction pattern is shown in Figure 29b. For the diffraction pattern simulated at a dose of 1 electron per Å^2^, the angular-averaged radial profile is shown in Figure 28c; here the intensity rapidly decreases and reaches a threshold of 1 cpp at *k* = 0.01 Å^−1^ which corresponds to a resolution of about 10 nm. At each radiation dose, the simulated diffraction pattern distribution was converted to integer numbers (to mimic cpp), the angular-averaged radial profile was extracted, the position of k corresponding to a threshold of 1 cpp was determined and the resolution corresponding to that value of k was estimated. The resulting plot is shown in Figure 29d.

A comparison of the results shown in Figure 28c and Figure 29d indicates that to achieve the same resolution, the radiation dose required by CDI is roughly a thousand times larger than that for in-line holography.

**Figure 30:**
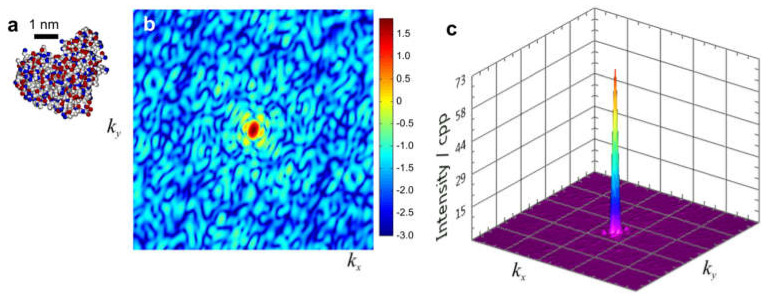
Simulated electron diffraction pattern of a single lysozyme molecule. (**a**) Structure of the lysozyme molecule. (**b**,**c**) simulated diffraction pattern with a radiation dose of 20 electrons per Å^2^ in 2D and 3D representations, respectively; here *k_x_* and *k_y_* range from −0.5 Å^−1^ to 0.5 Å^−1^, corresponding to a resolution at the rim of the diffraction pattern of 2 Å. The maximum of intensity is 73 cpp. Diffraction pattern (DP) in (**b**) is shown as log_10_(DP).

To provide an example of a more realistic sample, the diffraction pattern of a single lysozyme molecule was calculated at a radiation dose of 20 e/Å^2^ with the multi-slice simulation protocol provided in Appendix A. The results are shown in Figure 30. The maximum intensity in the diffraction pattern is seen at the center and is 73 cpp; the intensity rapidly decreases and reaches a threshold of 1 cpp at *k* = 0.035 Å^−1^ which corresponds to a resolution of about 2.85 nm. However, the intensity distribution in the central region is usually not acquired in an experiment, because of intense direct beam. Similar simulated diffraction patterns of individual lysozyme molecules were presented by Neutze et al. in a paper that addressed the possibility of single molecule diffraction with X-pulses from a free electron laser in a diffract-and-destroy experiment [129].

In summary, although CDI offers the theoretical possibility of recording high-resolution information, radiation damage limits the detected cpp in the diffraction pattern of a single molecule in practice, making it unsuitable to apply an iterative phase retrieval reconstruction routine to these diffraction patterns. To achieve a sufficiently strong signal for structure retrieval, other strategies such as averaging over thousands of diffraction patterns must be applied [130]. 

### 10.2. Low vs. High-Energy Electrons

Imaging with high-energy electrons is much more easily accessible, since high-energy electrons are employed in conventional TEMs and TEMs equipped with biprism(s) for off-axis holography experiments. Low-energy electron microscopes that operate in in-line holographic or point projection imaging regimes are self-built, and are not commercially available. High-energy electrons exhibit a relatively large IMFP, which allows for the imaging of materials tens of nanometers thick, while low-energy electrons can only image samples that are a few nanometers thick. Low-energy electrons are highly sensitive to potential distributions in an electron microscope, which causes artefactual deflection of the reference electron beam (the biprism effect) and complicates data analysis and evaluation of the sample structure [88,131]. To ensure an undisturbed reference wave, the sample needs to be placed onto an equipotential surface, for example graphene [92,93], in in-line low-energy electron holography. On the other hand, their high sensitivity to local potentials makes low-energy electrons the perfect type of radiation for studying 2D materials such as graphene and van der Waals structures [9,74,94,132]. Moreover, low-energy electrons can be employed for mapping unoccupied band structure of freestanding 2D materials, such as graphene by angle-resolved low-energy electron transmission measurements realized in in-line holography mode [133].

## Figures and Tables

**Table 1:** Advantages and disadvantages of in-line holography (defocus imaging) and CDI.

	In-Line Holography	CDI
Finding the sample in the microscope when imaging	Easy when imaging with widely expanded spherical wave (+)	Difficult when imaging with narrow collimated beam (−)
Phase information	Available from the recorded intensity (+)	Lost from the recorded intensity (−)
Reconstruction procedure	“One-step” reconstruction by calculating back-propagation integral (+)	Iterative reconstruction
Reconstructed information	*z*-information is available and a "three-dimensional" reconstruction is possible (+)	Reconstructed distribution is always a projection of the sample onto one plane (−)
Stability of the recorded image	Any lateral shift of the sample results in a lateral shift of the entire hologram (−)	Invariant to lateral shifts of the sample (+)
Resolution	Low resolution due to lateral and axial vibrations (−)	High resolution (+)

## Appendix A

The diffraction pattern of a single lysozyme molecule was calculated at a radiation dose of 20 e/Å^2^ with the following multi-slice simulation protocol:

1. Lysozyme atomic coordinates were downloaded from PDB 253L [134], and hydrogen atoms were added using Chimera software.
2. The sequence of atoms was re-arranged in order of increasing z-coordinate, and atoms were numbered as a1, a2 etc.
3. An incident plane wave with unit amplitude was assumed, $u_{1}(x_{1},y_{1},z_{1})=1.$
4. The coordinates of the first atom a1 were read from the text file as $\left(x_{1}^{\left(1\right)},y_{1}^{\left(1\right)},z_{1}\right)$.
5. The transmission function in plane at $z_{1}$ was calculated as $t_{1}(x_{1},y_{1},z_{1})=\exp\left[i\sigma v_{z}(x_{1},y_{1})\right]$, where σ is the interaction parameter at 200 keV and $v_{z}(x_{1},y_{1})$ is the projected potential of atom a1, calculated from the tabulated parameters corresponding to the chemical elements as described in reference [8].
6. The exit wave in the plane $\left(x_{1},y_{1},z_{1}\right)$ was calculated as ${u^{\prime }}_{1}(x_{1},y_{1},z_{1})=u_{1}(x_{1},y_{1},z_{1})t_{1}(x_{1},y_{1},z_{1}).$
7. The z-coordinate of the next atom a2 was read as $z_{2}$, and the distance $\Delta z=z_{2}-z_{1}$ was calculated.
8. The wave function ${u^{\prime }}_{1}(x_{1},y_{1},z_{1})$ was propagated for Δz using the angular spectrum method [77]. The resulting wavefront was $u_{2}(x_{2},y_{2},z_{2})$.

The wave function was propagated through the sample, atom by atom, by repeating steps 4 to 8 until the electron wave had propagated through all the atoms. Finally, the diffraction pattern was calculated as square of the amplitude of the FT of the exit wave.
